# On Berman Functions

**DOI:** 10.1007/s11009-023-10059-6

**Published:** 2024-01-05

**Authors:** Krzysztof Dȩbicki, Enkelejd Hashorva, Zbigniew Michna

**Affiliations:** 1 Mathematical Institute, University of Wrocław, pl. Grunwaldzki 2/4, 50-384 Wrocław, Poland; 2 Department of Actuarial Science, University of Lausanne, Chamberonne, 1015 Lausanne, Switzerland; 3 Department of Operations Research and Business Intelligence, Wrocław University of Science and Technology, 50-370 Wrocław, Poland

**Keywords:** Berman functions, Pickands constants, Max-stable random fields, Simulations, Primary 60G15, Secondary 60G70

## Abstract

Let $Z(t)= \exp \left( \sqrt{ 2} B_H(t)- \left|t \right|^{2H}\right) , t\in \mathbb {R}$ with $B_H(t),t\in \mathbb {R}$ a standard fractional Brownian motion (fBm) with Hurst parameter $H \in (0,1]$ and define for *x* non-negative the Berman function

$$\begin{aligned} \mathcal {B}_{Z}(x)= \mathbb {E} \left\{ \frac{ \mathbb {I} \{ \epsilon _0(RZ) > x\}}{ \epsilon _0(RZ)}\right\} \in (0,\infty ), \end{aligned}$$

where the random variable *R* independent of *Z* has survival function $1/x,x\geqslant 1$ and

$$\begin{aligned} \epsilon _0(RZ) = \int _{\mathbb {R}} \mathbb {I}{\left\{ RZ(t)> 1\right\} }{dt} . \end{aligned}$$

In this paper we consider a general random field (rf) *Z* that is a spectral rf of some stationary max-stable rf *X* and derive the properties of the corresponding Berman functions. In particular, we show that Berman functions can be approximated by the corresponding discrete ones and derive interesting representations of those functions which are of interest for Monte Carlo simulations presented in this article.

## Introduction

In the study of sojourns of rf’s in a series of papers by Berman, see e.g., (1982; 1992) a key random variable (rv) and a related constant appear. Specifically, let $Z(t)= \exp ( \sqrt{ 2} B_{{H}}(t)- \left|t \right|^{2 H}), t\in \mathbb {R},$ with $B_{H}$ a fractional Brownian motion (fBm) with Hurst parameter $H\in (0,1]$, that is a centered Gaussian process with stationary increments, $Var(B_H(t))=\left|t \right|^{2H},t\in \mathbb {R}$ and continuous sample paths. In view of Berman (1992, Thm 3.3.1, Eq. (3.3.6)) the following rv (hereafter $\mathbb {I}{\left\{ \cdot \right\} }$ is the indicator function)

$$\begin{aligned} \epsilon _0(RZ) = \int _{\mathbb {R}} \mathbb {I}{\left\{ RZ(t)> 1\right\} } dt \end{aligned}$$

plays a crucial role in the analysis of extremes of Gaussian processes. Throughout this paper *R* is a 1-Pareto rv ($\ln R$ is unit exponential) independent of any other random element.

The distribution function of $\epsilon _0(RZ)$ is known only for $H\in \{1/2,1\}$. For $H=1$ as shown in Berman (1992, Eq. (3.3.23)) $\epsilon _0(RZ)$ has probability density function (pdf) $x^2 e^{- x^2/2}/(2 \sqrt{ \pi }),x>0$, whereas for $H=1/2$ its pdf is calculated in Berman (1992, Eq. (5.6.9)).

The so-called Berman function defined for all $x\geqslant 0$ (see Berman (1992, Eq. (3.0.2))) given by

1.1 $$\begin{aligned} \mathcal {B}_{Z}(x) =\mathbb {E}{\left\{ \frac{ \mathbb {I}{\left\{ \epsilon _0(RZ) > x\right\} }}{ \epsilon _0(RZ)}\right\} }\in (0,\infty ) \end{aligned}$$

appears also in Berman (1992, Thm 3.3.1, Eq. (3.3.6)).

An important property of the Berman function is that for $x=0$ it equals the *Pickands* constant, see (Berman 1992, Thm 10.5.1) i.e., $\mathcal {B}_{Z }(0) = \mathcal {H}_{Z },$ where $\mathcal {H}_{Z }$ is the so called *generalised Pickands* constant

$$\begin{aligned} \mathcal {H}_{Z } = \lim _{T \rightarrow \infty } \frac{1}{T} \mathbb {E}{\left\{ \sup _{ t\in [0,T]} Z (t)\right\} }. \end{aligned}$$

This fact is crucial since $\mathcal {B}_{Z }(0)$ is the first known expression of $\mathcal {H}_{Z }$ in terms of an expectation, which is of particular usefulness for simulation purposes, see Falk et al. (2010), Dieker and Yakir (2014), and Hüsler and Piterbarg (2017) for details on classical Pickands constants.

Besides, Berman’s representation of Pickands constant yields tight lower bounds for $\mathcal {H}_{Z }$, see Dȩbicki et al. (2019, Thm 1.1). As shown in Dȩbicki et al. (2019) for all $x \geqslant 0$

$$\begin{aligned} \mathcal {B}_{Z }(x) = \lim _{T \rightarrow \infty } \frac{1}{T} \mathcal {B} ([0,T], x) , \quad \mathcal {B}_{Z } ([0,T], x ) := \int _0^\infty \mathbb {P}{\left\{ \int _{0}^T \mathbb {I}(Z (t)> s) dt > x \right\} } ds. \end{aligned}$$

Motivated by the above definition, in this contribution we shall introduce the Berman functions for given $\delta \geqslant 0$ with respect to some non-negative rf $Z(t),t\in \mathbb {R}^d,d\geqslant 1$ with càdlàg sample paths (see e.g., Janson (2020), and Bladt et al. (2022) for the definition and properties of generalised càdlàg functions) such that

1.2 $$\begin{aligned} \mathbb {E}{\left\{ Z (t) \right\} }=1, \quad t\in \mathbb {R}^d. \end{aligned}$$

Specifically, for given non-negative $\delta , x$ define

$$\begin{aligned} \mathcal {B}_{Z }^\delta (x):=\lim _{T\rightarrow \infty }\frac{1}{T^{d}} \mathcal {B}_{Z }^\delta ([0,T]^d \cap \delta \mathbb {Z}^d, x), \end{aligned}$$

where

$$\begin{aligned} \mathcal {B}_{Z }^\delta ([0,T]^d \cap \delta \mathbb {Z}^d, x):= \int _0^\infty \mathbb {P}{\left\{ \int _{[0,T]^d \cap \delta \mathbb {Z}^d} \mathbbm {I}(Z (t)> s) \lambda _\delta (dt) > x \right\} }ds. \end{aligned}$$

Here $\lambda _0(dt)=\lambda (dt)$ is the Lebesgue measure on $\mathbb {R}^d$, $0 \mathbb {Z}^d=\mathbb {R}^d$ and $\lambda _\delta (dt)/\delta ^d$ is the counting measure on $\delta \mathbb {Z}^d$ if $\delta >0$. Hence $\mathcal {B}_{Z }^\delta (x),\delta >0$ is the discrete counterpart of $\mathcal {B}_{Z }(x)$ and $\mathcal {B}_{Z }^0(x)=\mathcal {B}_{Z }(x)$.

In general, in order to be well-defined for the function $\mathcal {B}_{Z}^\delta (x), x\geqslant 0$ some further restriction on the rf *Z* are needed. A very tractable case for which we can utilise results from the theory of max-stable stationary rf’s is when *Z* is the spectral rf of a stationary max-stable rf $X(t), t\in \mathbb {R}^d$, see (2.1) below.

An interesting special case is when $\ln Z(t)$ is a Gaussian rf with trend equal to the half of its variance function having further stationary increments. We shall show in Lemma 4.3 that for such *Z* the corresponding Berman function $\mathcal {B}_{Z }(x)$ appears in the tail asymptotic of the sojourn of a related Gaussian rf.

Organisation of the rest of the paper. In Section 2 we first present in Theorem 2.1 a formula for Berman functions and then in Corollary 2.3 and Proposition 3.1 we show some continuity properties of those functions. In Theorem 2.5 and Lemma 2.4 we present two representations for Berman functions and discuss conditions for their positivity. Section 3 is dedicated to the approximation of Berman functions focusing on the Gaussian case. All the proofs are postponed to Section 4.

## Main Results

Let the rf $Z(t), t\in \mathbb {R}^d$ be as above defined in the non-atomic complete probability space $(\Omega , \mathcal {F}, \mathbb {P})$. Let further $X(t),t\in \mathbb {R}^d$ be a max-stable stationary rf, which has spectral rf *Z* in its de Haan representation (see e.g., de Haan (1984), Kulik and Soulier (2020))

2.1 $$\begin{aligned} X(t)= \max _{i\geqslant 1} \Gamma _i^{{-1}} Z^{(i)}(t), \quad t\in \mathbb {R}^d. \end{aligned}$$

Here $\Gamma _i= \sum _{k=1}^i \mathcal {V}_k$ with $\mathcal {V}_k, k\geqslant 1$ mutually independent unit exponential rv’s being independent of $\{Z^{(i)}\}_{i=1}^\infty$ which are independent copies of *Z*. For simplicity we shall assume that the marginal distributions of the rf *X* are unit Fréchet (equal to $e^{-1/x },x>0$) which in turn implies $\mathbb {E}{\left\{ Z (t)\right\} } =1$ for all $t\in \mathbb {R}^d$.

Suppose further that for all $T>0$

2.2 $$\begin{aligned} \mathbb {E}{\left\{ \sup _{t\in [0,T]^d} Z (t)\right\} }< \infty \end{aligned}$$

and *Z* has almost surely sample paths on the space *D* of non-negative càdlàg functions $f: \mathbb {R}^d \mapsto [0, \infty )$ equipped with Skorohod’s $J_1$-topology. We shall denote by $\mathcal {D}=\sigma (\pi _t, t\in T_0)$ the $\sigma$-field generated by the projection maps $\pi _t: \pi _t f= f(t), f\in D$ with $T_0$ a countable dense subset of $\mathbb {R}^d$. In view of Hashorva (2018, Thm 6.9) with $\alpha =1, L=B^{-1}$, see also Planinić and Soulier (2018, Eq. (5.2)) the stationarity of *X* is equivalent with

2.3 $$\begin{aligned} \mathbb {E}{\left\{ Z (h) F(Z)\right\} } = \mathbb {E}\{ Z (0) F(B^h Z)\}, \quad \forall h\in \mathbb {R}^d \end{aligned}$$

valid for every measurable functional $F: D \rightarrow [0,\infty ]$ such that $F(cf)= F(f)$ for all $f\in D, c>0$. Here we use the standard notation $B^h Z(\cdot )= Z(\cdot -h), h\in \mathbb {R}^d$.

We shall suppose next without loss of generality (see Hashorva (2021, Lem 7.1)) that

2.4 $$\begin{aligned} \mathbb {P}{\left\{ \sup _{t\in \mathbb {R}^d} Z(t) >0\right\} }=1. \end{aligned}$$

Under the assumption that *X* is stationary $\mathcal {B}_{Z }^\delta (x)$ is well-defined for all $\delta ,x$ non-negative as we shall show below. We note first that, see e.g., Dȩbicki et al. (2019, 2022)

$$\begin{aligned} \lim _{T \rightarrow \infty } {\frac{1}{T^{d}}}\mathcal {B}_{Z}^\delta ([0,T]^{d}\cap \delta \mathbb {Z}^{{\delta }} ,0)=\mathcal {B}_{Z}^\delta (0) =\mathcal {H}_{Z}^\delta \in (0, \infty ), \end{aligned}$$

where $\mathcal {H}_{Z}^\delta$ is the discrete counterpart of the classical Pickands constant $\mathcal {H}_{Z}=\mathcal {H}_{Z}^0$. Hence for any $x>0$ we have

$$\begin{aligned} \mathcal {B}_{Z}^\delta (x) \leqslant \mathcal {H}_{ Z }^\delta < \infty . \end{aligned}$$

Set below for $\delta >0$

$$\begin{aligned} S_\delta =S_\delta (Z)=\int _{ \delta \mathbb {Z}^d} Z (t) \lambda _\delta (dt)=\delta ^d \sum _{ t\in \delta \mathbb {Z}^d} Z (t) \end{aligned}$$

and let $S_0=S_0(Z)=\int _{ \mathbb {R}^d} Z (t) \lambda (dt)$. In view of (2.4) we have that $S_0>0$ almost surely. Since we do not consider the case $\delta >0$ and $\delta =0$ simultaneously, we can assume that $S_\delta >0$ almost surely (we can construct a spectral rf *Z* for *X* that guarantees this, see Hashorva (2021, Lem 7.3)).

In view of Dȩbicki et al. (2022, Cor 2.1) if $\mathbb {P}{\left\{ S_0=\infty \right\} } =1$, then $\mathcal {H}_{ Z }=0$ implying

$$\begin{aligned} \mathcal {B}_{ Z }^\delta (x)=\mathcal {H}_{ Z }=0, \quad \forall \delta , x\geqslant 0. \end{aligned}$$

The next result states the existence and the positivity of Berman functions presenting further a tractable formula that is useful for simulations of those functions.

### Theorem 2.5

If $\mathbb {P}{\left\{ S_0=\infty \right\} } <1$, then for any $\delta , x$ non-negative constants we have

2.5 $$\begin{aligned} \mathcal {B}_{ Z }^\delta (x) = \int _0^\infty \mathbb {E}{\left\{ \frac{ Z (0)}{ S_{{\delta }}} \mathbbm {I} \Bigl ( \int _{\delta \mathbb {Z}^d} \mathbbm {I}( Z (t)> s) \lambda _\delta (dt) > x \Bigr ) \right\} }\lambda (ds) < \infty . \end{aligned}$$

Moreover, (2.5) holds substituting $S_\delta$ by $S_\eta$, where $\eta >0$ if $\delta =0$ and $\eta =k \delta , k\in \mathbb {N}$ if $\delta >0$, provided that

2.6 $$\begin{aligned} \{ {S_0(Z)} < \infty \} \subset \{ S_\eta (B^r Z) \in (0,\infty ) \}, \quad \forall r\in \delta \mathbb {Z}^d \end{aligned}$$

almost surely.

### Remark 2.2

(i) If $x=0$, then we retrieve the results of Dȩbicki et al. (2022, Prop 2.1).
(ii) As shown in Dȩbicki et al. (2022) condition (2.6) holds in the particular case that $Z(t)>0,t\in \mathbb {R}^d$ almost surely.
(iii) One example for *Z*, see for instance Dȩbicki et al. (2022) is taking
  $$\begin{aligned} Z(t)= \exp ( V(t)- \sigma ^2_V(t)/2)), \quad t\in \mathbb {R}^d, \end{aligned}$$
  where $V(t),t\in \mathbb {R}^d$ is a centered Gaussian rf with almost surely continuous trajectories and stationary increments, $\sigma ^2_V(t)=Var(V(t))$ and $\sigma _V(0)=0$. For this case $Z(t)>0,t\in \mathbb {R}^d$ almost surely, condition (2.6) is satisfied and (2.5) reads
  2.7 $$\begin{aligned} \mathcal {B}_{ Z }^\delta (x) =\int _0^\infty \mathbb {E}{\left\{ \frac{1}{ S_{{\delta }}} \mathbbm {I} \Bigl ( \int _{\delta \mathbb {Z}^d} \mathbbm {I}( V(t) - \sigma ^2_V(t)/2> \ln s) \lambda _\delta (dt) > x \Bigr ) \right\} }\lambda (ds) < \infty . \end{aligned}$$

### Corollary 2.3

If *Z* has almost surely continuous trajectories, then for all $x_0\geqslant 0$

2.8 $$\begin{aligned} \lim _{ x\rightarrow x_0} \mathcal { B}_{ Z }^ 0(x)= \mathcal { B}_{ Z }^ 0(x_0). \end{aligned}$$

Define next a probability measure $\mu$ on $\mathcal {D}$ by

2.9 $$\begin{aligned} \mu (A)= \mathbb {E}{\left\{ Z (0)\mathbb {I}{\left\{ Z/Z(0) \in A\right\} }\right\} }, \quad A \in \mathcal {D}. \end{aligned}$$

Let $\Theta$ be a rf with law $\mu$. By the definition, $\Theta$ has also càdlàg sample paths and since *D* is Polish, in view of Varadarajan (1958, Lem. p. 1276) we can assume that $\Theta$ is defined in the same probability space as *Z*. Recall that $\lambda _\delta (dt)/\delta ^d$ is the counting measure on $\delta \mathbb {Z}^d$ if $\delta >0$ and $\lambda _0$ is the Lebesgue measure on $\mathbb {R}^d$. Since we can rewrite (2.5) as

2.10 $$\begin{aligned} \mathcal { B}_{ Z }^ \delta (x) = \int _0^\infty \mathbb {E}{\left\{ \frac{1}{ S_{{\delta }}(\Theta ) } \mathbbm {I} \Bigl ( \int _{\delta \mathbb {Z}^d} \mathbbm {I}( {\Theta } (t)> s) \lambda _\delta (dt) > x \Bigr ) \right\} }\lambda (ds) < \infty , \end{aligned}$$

where

$$\begin{aligned} S_{{\delta }}(\Theta )= \int _{{\delta \mathbb {Z}^d}} \Theta (t) \lambda _\delta (dt) \end{aligned}$$

and the law of $\Theta$ is uniquely determined by the law of the max-stable stationary rf *X* and does not depend on the particular choice of *Z*, see Hashorva (2018, Lem A.1), hence if $Z_*$ is another spectral rf for *X*, then

2.11 $$\begin{aligned} \mathcal { B}_{ Z }^ \delta (x)= \mathcal { B}_{{Z_*}}^ \delta (x) \end{aligned}$$

for all $\delta \geqslant 0$. Assume next that $\mathbb {P}{\left\{ S_0< \infty \right\} }=1$ and let

2.12 $$\begin{aligned} Z_*(t)= (p(T))^{-1}B^T {\bar{Q}_\delta (t)}, \quad t\in \delta \mathbb {Z}^d \end{aligned}$$

be a spectral rf of the max-stable rf $X_\delta (t)= X(t), t\in \delta \mathbb {Z}^d$, where $\bar{Q}_\delta$ is independent of a rv *T*, which has pdf $p(s)>0, \,s\in {\delta \mathbb {Z}^d}$. We choose *p* to be continuous when $\delta =0$. In view of Dȩbicki and Hashorva (2020, Thm 2.3) one possible construction is

$$\begin{aligned} \bar{Q}_\delta (t)= c\frac{\Theta (t)}{ S_\delta (\Theta )} , \quad t\in \delta \mathbb {Z}^d, \end{aligned}$$

with $c=1$ if $\delta =0$ and $c=\delta ^d$ otherwise. Set below $Q_\delta = \bar{Q}_\delta /c$.

### Lemma 2.4

(i) If $\mathbb {P}{\left\{ S_0< \infty \right\} }=1$, then for $Q_\delta$ as above and all $\delta ,x$ non-negative we have
  2.13 $$\begin{aligned} \mathcal { B}_{ Z }^ \delta (x) = \int _0^\infty \mathbb {P}{\left\{ \int _{\delta \mathbb {Z}^d} \mathbbm {I}( Q_\delta (t)> s) \lambda _\delta (dt) > x \right\} }\lambda (ds) < \infty . \end{aligned}$$
(ii) If $\mathbb {P}{\left\{ S_0< \infty \right\} }>0$, then with $V(t)= Z(t) |S_0 < \infty$ for all $\delta ,x$ non-negative we have
  2.14 $$\begin{aligned} \mathcal { B}_{ Z }^ \delta (x) = \mathbb {P}{\left\{ S_0(\Theta )< \infty \right\} } \mathcal { B}_{V}^ \delta (x) < \infty . \end{aligned}$$

Let in the following $Y(t)= R \Theta (t)$ with *R* a 1-Pareto rv with survival function $1/x, x \geqslant 1$ independent of $\Theta$ and set hereafter

$$\begin{aligned} \epsilon _\delta (Y)= \int _{\delta \mathbb {Z}^d} \mathbb {I}{\left\{ Y(t)> 1\right\} } \lambda _\delta (dt). \end{aligned}$$

Recall that when $\delta =0$ we interpret $\delta \mathbb {Z}^d$ as $\mathbb {R}^d$. We establish below the Berman representation (1.1) for the general setup of this paper.

### Theorem 3.2

If $\mathbb {P}{\left\{ S_0=\infty \right\} } <1$, then for all $\delta , x$ non-negative

2.15 $$\begin{aligned} \mathcal { B}_{ Z }^\delta (x) = \mathbb {E}{\left\{ \frac{ \mathbb {I}{\left\{ \epsilon _\delta (Y )> x \right\} }}{\epsilon _\delta (Y )} \right\} } < \infty . \end{aligned}$$

### Corollary 2.6

Under the conditions of Theorem 2.5 we have that $\epsilon _0 (Y )$ has a continuous distribution if *Z* has almost surely continuous trajectories. Moreover, $\mathcal { B}_{ Z }^\delta (x) >0$ for all $x\geqslant 0$ such that $\mathbb {P}{\left\{ \epsilon _\delta (Y )>x\right\} } >0$.

### Proposition 2.7

For all $\delta \geqslant 0$ and $x>0$ we have

2.16 $$\begin{aligned} \frac{\mathbb {P}{\left\{ \epsilon _\delta (Y)>x\right\} }^2}{\mathbb {E}{\left\{ \epsilon _\delta (Y)\right\} }} \leqslant \mathcal { B}_{ Z }^\delta (x) \leqslant x^{-1}\mathbb {P}{\left\{ \epsilon _\delta (Y)\geqslant x\right\} }. \end{aligned}$$

### Remark 2.8

(i) If $x=0$, the lower bound in (2.16) holds with 1 in the numerator, see Dȩbicki et al. (2019), and Hashorva (2021).
(ii) If $\mathbb {E}{\left\{ \epsilon ^p_\delta (Y)\right\} }$ is finite for some $p>0$, then combination of the upper bound in (2.16) with the Markov inequality gives the following upper bound
  2.17 $$\begin{aligned} \mathcal { B}_{ Z }^\delta (x) \leqslant x^{-p-1}\mathbb {E}{\left\{ \epsilon ^p_\delta (Y)\right\} }, \quad x>0. \end{aligned}$$
(iii) If $\mathbb {E}{\left\{ \epsilon _\delta (Y)\right\} }<\infty$ and $\int _0^\infty e^{sx}(\mathcal { B}_{ Z }^\delta (x))^{1/2}dx<\infty$, then it follows that for all $s>0$
  $$\begin{aligned} \mathbb {E}{\left\{ e^{s\epsilon _\delta (Y)}\right\} }\leqslant 1+s(\mathbb {E}{\left\{ \epsilon _\delta (Y)\right\} })^{1/2}\int _0^\infty e^{sx}(\mathcal { B}_{ Z }^\delta (x))^{1/2}dx. \end{aligned}$$
(iv) Since $Y= R\Theta$, we can calculate in case of known $\Theta$ the expectation of $\epsilon _\delta (Y)$ as follows
  $$\begin{aligned} \mathbb {E}{\left\{ \epsilon _\delta (Y)\right\} }= & {} \int _{\delta \mathbb {Z}^d} \mathbb {P}{\left\{ R\Theta (t)> 1\right\} } \lambda _\delta (dt) =\int _1^\infty \int _{\delta \mathbb {Z}^d}\mathbb {P}{\left\{ \Theta (t)> 1/r\right\} }\lambda _\delta (dt)r^{-2}dr. \end{aligned}$$
  If $Z(t)= \exp ( V(t)- \sigma ^2_V(t)/2)), t\in \mathbb {R}^d$ is as in Remark 2.2, Item (iii), then in view of Dȩbicki et al. (2019, Lem 5.4), and Hashorva (2021, Eq. (5.3)) we have
  2.18 $$\begin{aligned} \begin{aligned} \mathbb {E}{\left\{ \epsilon _\delta (Y)\right\} }&= \int _{\delta \mathbb {Z}^d}\int _1^\infty {\Psi }\left( \frac{\sigma _V(t)}{2}-\frac{\ln r}{\sigma (t)}\right) r^{-2}\lambda _\delta (dt)dr \\&= 2 \int _{t\in \delta \mathbb {Z}^d} {\Psi }(\sigma _V(t)/2)\lambda _\delta (dt), \end{aligned} \end{aligned}$$
  where $\Psi$ is the survival function of an *N*(0, 1) rv.

## Approximation of $\mathcal {B}_{Z }^\delta (x)$ and its Behaviour for Large *x*

We show first that $\mathcal { B}_{ Z }=\mathcal { B}_{ Z }^{0}$ can be approximated by considering $\mathcal { B}_{ Z }^{ \delta }(x)$ and letting $\delta \downarrow 0$.

### Proposition 3.1

For all $x\geqslant 0$ we have that

$$\begin{aligned} \lim _{\delta \downarrow 0} \mathcal { B}_{ Z }^{ \delta } (x) = \mathcal { B}_{ Z }^{0} (x) . \end{aligned}$$

We note in passing that for $x=0$ we retrieve the approximation for Pickands constants derived in Dȩbicki et al. (2022). An approximation of $\mathcal { B}_{ Z }^{ \delta }(x)$ can be obtained by letting $T\rightarrow \infty$ and calculating the limit of

$$\begin{aligned} \frac{\mathcal {B}^\delta _{Z }([0,T]^d\cap \delta \mathbb {Z}^d,x)}{ T^d}. \end{aligned}$$

For such an approximation we shall discuss the rate of convergence to $\mathcal { B}_{ Z }^{ \delta }(x)$ assuming further that

$$\begin{aligned} Z(t)=\exp \left( V(t)- \frac{\sigma ^2_V(t)}{2}\right) , \quad t\in \mathbb {R}^d \end{aligned}$$

is as in Remark 2.2, Item (iii).

**A1** $\sigma ^2_V(t)$ is a continuous and strictly increasing function, and there exists $\alpha _0\in (0,2]$ and $A_0\in (0,\infty )$ such that

$$\begin{aligned} \limsup _{\Vert t\Vert \rightarrow 0}\frac{\sigma ^2_V(t)}{\Vert t\Vert ^{\alpha _0}}\leqslant A_0, \end{aligned}$$

where $\Vert \cdot \Vert$ is the Euclidean norm.

**A2** There exists $\alpha _\infty \in (0,2]$ such that

$$\begin{aligned} \liminf _{\Vert t\Vert \rightarrow \infty } \frac{\sigma ^2_V(t)}{\Vert t\Vert ^{\alpha _\infty }}>0. \end{aligned}$$

The following theorem constitutes the main finding of this section.

### Theorem 2.1

Under **A1-A2** we have for all $\delta , x$ non-negative and $\lambda \in (0,1)$

3.1 $$\begin{aligned} \lim _{T\rightarrow \infty }\left| \mathcal { B}_{ Z }^\delta (x)-\frac{\mathcal { B}_{ Z }^\delta ([0,T]^d\cap \delta \mathbb {Z}^d,x)}{T^d}\right| T^\lambda =0. \end{aligned}$$

### Remark 3.3

(i) For $x=0$ the rate of convergence in (3.1) agrees with the findings in Dȩbicki (2005).
(ii) The range of the parameter $\lambda \in (0,1)$ in Theorem 3.2 cannot be extended to $\lambda \geqslant 1$. Indeed, following Ling and Zhang (2020), for $V(t)=\sqrt{2}B_{1}(t)$, $\delta =0$, $T>x$ and $d=1$ we have
  $$\begin{aligned} \mathcal { B}_{ Z }([0,T],x)= 2\Psi (x/\sqrt{2})+\sqrt{2}(T-x)\varphi (x/\sqrt{2}) \end{aligned}$$
  implying
  3.2 $$\begin{aligned} \mathcal { B}_{ Z }(x)=\sqrt{2}\varphi (x/\sqrt{2}), \end{aligned}$$
  where $\varphi (\cdot )$ is the pdf of an *N*(0, 1) rv. Consequently, we have
  $$\begin{aligned} \lim _{T\rightarrow \infty } \left| \mathcal { B}_{ Z }(x)-\frac{\mathcal { B}_{ Z }([0,T],x)}{T} \right| T ={|2\Psi (x/\sqrt{2}) -\sqrt{2}x \varphi (x/\sqrt{2})|} >0. \end{aligned}$$

In the rest of this section we focus on $d=1$ log-Gaussian case. In view of (3.2) for some finite positive constant *C*

$$\begin{aligned} \ln (\mathcal { B}_{ Z }^\delta (x)) \sim -{ C} \sigma ^2_V(x), \quad x\rightarrow \infty . \end{aligned}$$

The next result gives logarithmic bounds for $\mathcal { B}_{ Z }^\delta (x)$ as $x\rightarrow \infty$ that supports this hypothesis.

### Proposition 3.4

Suppose that $d=1$ and *V* satisfies **A1-A2**. Then

$$\begin{aligned} \liminf _{x\rightarrow \infty }\frac{\ln (\mathcal { B}_{ Z }^\delta (x))}{\sigma ^2_V(x/2)}\geqslant -1 \end{aligned}$$

and

$$\begin{aligned} \limsup _{x\rightarrow \infty }\frac{\ln (\mathcal { B}_{ Z }^\delta (x))}{\sigma ^2_V(x/2)}\leqslant -\frac{3-2\sqrt{2}}{2}. \end{aligned}$$

**Table 1:** Values of $\mathbb {E}{\left\{ \epsilon _\delta (Y)\right\} }$ for $\delta =\{0,1,5,10\}$ and $V(t)=\sqrt{2}B_H(t)$

$\mathbb {E}{\left\{ \epsilon _\delta (Y)\right\} }\,\,\backslash \,\, H$	0.1	0.2	0.3	0.4	0.5
$\delta =0$	60480	72.216267	12.309822	5.866446	4
$\delta =1$	48824.040913	72.594979	12.632020	6.140195	4.232120
$\delta =5$	57667.986631	74.736598	14.803952	8.344951	6.474827
$\delta =10$	59291.12614	77.87128	18.22637	12.07630	10.54057

$\mathbb {E}{\left\{ \epsilon _\delta (Y)\right\} }\,\,\backslash \,\, H$	0.6	0.7	0.8	0.9	1
$\delta =0$	3.198992	2.777685	2.527405	2.366354	2.256758
$\delta =1$	3.395236	2.942920	2.665777	2.481422	2.351603
$\delta =5$	5.685059	5.295399	5.104008	5.026130	5.004070
$\delta =10$	10.09794	10.00788	10.00016	10	10

**Fig. 1:**
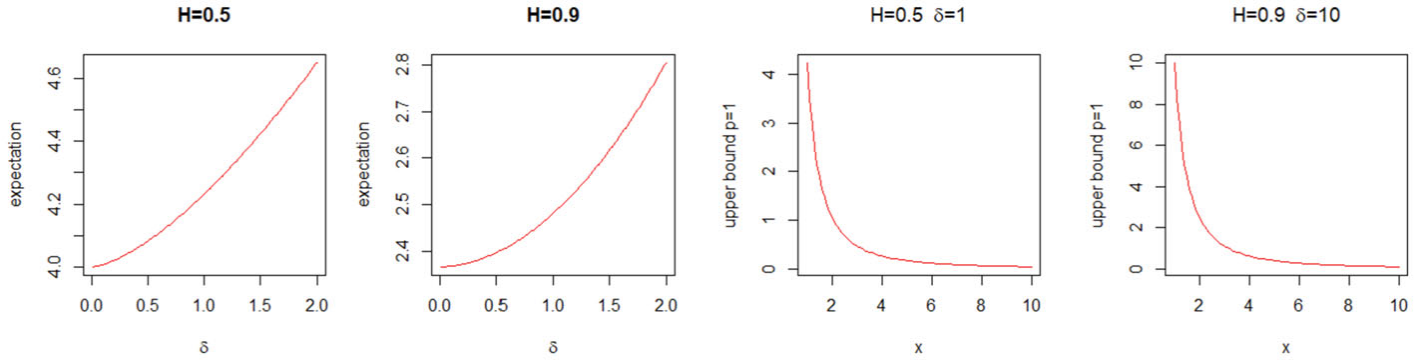
$\mathbb {E}{\left\{ \epsilon _\delta (Y)\right\} }$ as a function of $\delta \in [0,2]$ and $H=\{0.5, 0.9\}$ and the upper bound (2.17) with $p=1$ for Berman constants as a function of $x\in [1,10]$ for $H=0.5$, $\delta =1$ and $H=0.9$, $\delta =10$ where $V(t)=\sqrt{2}B_H(t)$

### Remark 3.5

(i) If we suppose additionally that $\sigma _V^2$ is regularly varying at $\infty$ with parameter $\alpha >0$, then it follows from Proposition 3.4 that
  $$\begin{aligned} - \frac{1}{2^{\alpha }}\leqslant \liminf _{x\rightarrow \infty }\frac{\ln (\mathcal { B}_{ Z }^\delta (x))}{\sigma ^2_V(x)}\leqslant \limsup _{x\rightarrow \infty }\frac{\ln (\mathcal { B}_{ Z }^\delta (x))}{\sigma ^2_V(x)}\leqslant -\frac{3-2\sqrt{2}}{2^{\alpha +1}}. \end{aligned}$$
(ii) If follows from the proof of Proposition 3.4 that under **A1-A2**
  $$\begin{aligned} - \frac{1}{2}\leqslant \liminf _{x\rightarrow \infty }\frac{\ln (\mathbb {P}{\left\{ \epsilon _\delta (Y)>x\right\} })}{\sigma ^2_V(x/2)}\leqslant \limsup _{x\rightarrow \infty }\frac{\ln (\mathbb {P}{\left\{ \epsilon _\delta (Y)>x\right\} })}{\sigma ^2_V(x/2)}\leqslant -\frac{3-2\sqrt{2}}{2}. \end{aligned}$$

**Table 2:** Estimation of $\mathcal { B}_{ Z } (x)$ for fBm $B_H$ and the half width of 95% confidence interval

$x\backslash H$	0.4	0.5	0.6	0.7
0	1.016664 ± 0.08504517	1.038244 ± 0.08105526	0.9336774 ± 0.08679748	0.8059135 ± 0.04206874
0.05	0.7319381 ± 0.02164056	0.7662378 ± 0.02001129	0.77076 ± 0.0188357	0.7419288 ± 0.01654269
0.1	0.6285574 ± 0.01509329	0.6975815 ± 0.01508846	0.7083258 ± 0.01388002	0.6965521 ± 0.01262112
0.2	0.5210044 ± 0.01018901	0.5855574 ± 0.01005615	0.6169511 ± 0.009730012	0.6341507 ± 0.009250254
0.4	0.3996749 ± 0.00644895	0.4631784 ± 0.006531524	0.5050474 ± 0.006421914	0.5339913 ± 0.006178146
0.5	0.3523235 ± 0.005388992	0.4228112 ± 0.005689292	0.4642559 ± 0.005635201	0.4931276 ± 0.005447861
1	0.2390332 ± 0.003261513	0.2833468 ± 0.00342165	0.3098104 ± 0.003471659	0.3418117 ± 0.003439146
2	0.1373955 ± 0.001904439	0.1493499 ± 0.002068819	0.1606709 ± 0.002168721	0.1731576 ± 0.002275878
5	0.04407294 ± 0.0008552848	0.0366231 ± 0.0008681481	0.02843742 ± 0.0008307949	0.02065171 ± 0.0007567421
6	0.03259252 ± 0.0007073306	0.02488601 ± 0.0006875452	0.01711836 ± 0.000618167	0.01001073 ± 0.0005032682

$x\backslash H$	0.8	0.9	0.999	
0	0.7146539 ± 0.01835475	0.6482967 ± 0.01264331	0.5644909 ± 0.00675525	
0.05	0.6972516 ± 0.01375789	0.641973 ± 0.01041449	0.5660913 ± 0.006183973	
0.1	0.670074 ± 0.01129947	0.6275897 ± 0.008966844	0.5638993 ± 0.005561737	
0.2	0.6143437 ± 0.008171438	0.5951561 ± 0.007041064	0.5601536 ± 0.004994959	
0.4	0.5503154 ± 0.005904937	0.5457671 ± 0.005230998	0.5397937 ± 0.004207075	
0.5	0.5079599 ± 0.005075071	0.5182281 ± 0.004643272	0.5306746 ± 0.003954409	
1	0.3673532 ± 0.003371701	0.3978701 ± 0.003232641	0.4387592 ± 0.002982276	
2	0.1824689 ± 0.002382227	0.1975992 ± 0.002493246	0.2061034 ± 0.00260955	
5	0.01204482 ± 0.0006104972	0.004497369 ± 0.0003907966	0.001105259 ± 0.0001991857	
6	0.003932873 ± 0.0003303923	0.0006936917 ± 0.0001440762	6.375708e-05 ± 4.42043e-05

**Table 3:** Estimation of $\mathcal { B}_{ Z } (x)$ for integrated Ornstein-Uhlenbeck process and the half width of 95% confidence interval

*x*	0	0.5	1
$\mathcal { B}_{Z}(x)$	0.5267956 ± 0.01817717	0.452556 ± 0.004676632	0.3482289 ± 0.003180162

*x*	1.5	2	2.5
$\mathcal { B}_{Z}(x)$	0.2621687 ± 0.002588018	0.1900299 ± 0.002216284	0.1376086 ± 0.001910763

*x*	3	4	5
$\mathcal { B}_{Z}(x)$	0.09881259 ± 0.00163841	0.05088893 ± 0.00116684	0.02715927 ± 0.0008278098

*x*	6	7	8
$\mathcal {B}_{Z}(x)$	0.01433577 ± 0.0005788133	0.007437053 ± 0.0003983809	0.003796336 ± 0.0002730899

*x*	9	10	11
$\mathcal { B}_{Z}(x)$	0.001998398 ± 0.0001906838	0.001205136 ± 0.0001414664	0.000631948 ± 9.837308e-05

*x*	12	13	14
$\mathcal {B}_{Z}(x)$	0.0003812784 ± 7.355149e-05	0.0001845301 ± 4.89203e-05	0.00010499 ± 3.593126e-05

*x*	15	16	17
$\mathcal { B}_{Z}(x)$	9.130422e-05 ± 3.276786e-05	2.426165e-05 ± 1.594309e-05	2.103512e-05 ± 1.463212e-05

### Example 3.6

Let $V(t)=\sqrt{2}B_H(t)$, with $H\leqslant 1$, i.e., $\sigma ^2_V(t)=2t^{2H}$. Then $\mathbb {E}{\left\{ \epsilon _0(Y)\right\} }=\frac{4^{1/(2H)+0.5}}{\sqrt{\pi }\Gamma (1/(2H)+0.5)}$, see Dȩbicki et al. (2019). For $\delta >0$ we use (2.18) to compute $\mathbb {E}{\left\{ \epsilon _\delta (Y)\right\} }$, see Table 1. The graph of $\mathbb {E}{\left\{ \epsilon _\delta (Y)\right\} }$ as a function of $\delta$ and the upper bound (2.17) with $p=1$ for Berman constants as a function of $x\in [1,10]$ are presented on Fig. 1.

We simulated Berman constant $\mathcal { B}_{ Z } (x)$ using estimator (2.15) for different *x* and *H* see Table 2. In our simulation we generated $N=20000$ trajectories by means of Davies-Harte algorithm on the interval $[-64, 64]$ with the step $e=1/2^9=0.001953125$. Since the sample paths of fractional Brownian motion are very torn by the negative correlation of increments for $H<0.5$ we cannot trust the simulation for *H* close to 0 and we estimated Berman constant for $H\ge 0.4$ (see the half width of 95% confidence interval in Table 2). Let us note that the estimator (2.15) for $x=0$ is different from the estimator of Pickands constant in Dieker and Yakir (2014). Compare our simulation for $x=0$ with the results of Dieker and Yakir (2014) for Pickands constant.

### Example 3.7

**Fig. 2:**
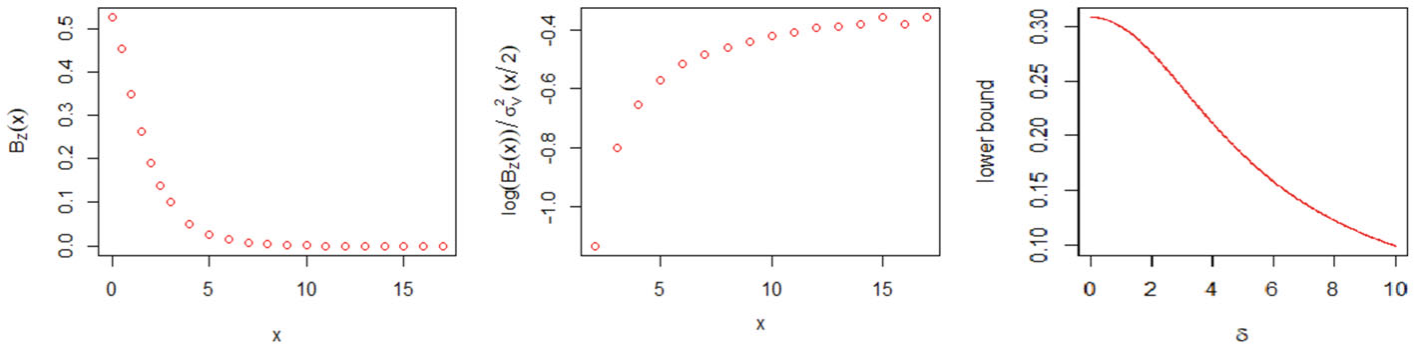
The graphs of $\mathcal { B}_{ Z }(x)$ and $\frac{\ln (\mathcal { B}_{ Z }(x))}{\sigma _V^2(x/2)}$ as function of *x* and the lower bound of Pickands constant as a function of $\delta$ for integrated Ornstein-Uhlenbeck process

Let *X*(*t*), $t\in \mathbb {R}$ be a stationary Ornstein-Uhlenbeck process, i.e., a centered Gaussian process with zero mean and covariance $\mathbb {E}{\left\{ X(t) X(s)\right\} }=\exp (-|t-s|),s,t\in \mathbb {R}$. Then the random process

$$\begin{aligned} V(t)= \left\{ \begin{array}{ll} \sqrt{2}\int _0^t X(s)ds &{}\text {if}\ t\geqslant 0\\ &{}\\ -\sqrt{2}\int _t^0 X(s)ds &{}\text {if}\ t<0 \end{array} \right. \end{aligned}$$

is Gaussian with stationary increments and variance $\sigma ^2_V(t)=4(|t|+e^{-|t|}-1$). Using (2.15) we simulated the Berman constant for $\delta =0$ and different *x*, see Table 3 and for $x=0$ and $\delta =\{0, 0.1, 0.2, 0.5, 1,2,5, 10\}$, see Table 4. We generated $N=20000$ trajectories with the step $e=10^{-5}$ on the interval $[-15, 15]$. In Fig. 2 we graphed $\mathcal { B}_{ Z }(x)$ and $\frac{\ln (\mathcal { B}_{ Z }(x))}{\sigma _V^2(x/2)}$ as function of *x* and we get that this ratio is asymptotically around $-0.4$. Note that according to Remark 3.5 it should be between $-0.5$ and $-0.04289322$.

**Table 4:** Estimation of $\mathcal { B}^{\delta }_{ Z }(0)$ for integrated Ornstein-Uhlenbeck process and the half width of 95% confidence interval

$\delta$	0	0.1
$\mathcal { B}^{\delta }_{ Z }(0)$	0.5267956 ± 0.01817717	0.5131973 ± 0.007850686

$\delta$	0.2	0.5
$\mathcal { B}^{\delta }_{ Z }(0)$	0.5126575 ± 0.007194036	0.4934096 ± 0.005746635

$\delta$	1	2
$\mathcal { B}^{\delta }_{ Z }(0)$	0.4484668 ± 0.00402201	0.3843544 ± 0.001995524

$\delta$	5	10
$\mathcal { B}^{\delta }_{ Z }(0)$	0.1908583 ± 0.0004065713	0.09984 ± 3.913749e-05

Using (2.18) we computed $\mathbb {E}{\left\{ \epsilon _\delta (Y)\right\} }$ and $1/\mathbb {E}{\left\{ \epsilon _\delta (Y)\right\} }$ for $\delta =\{0,0.1,0.2, 0.5,1,2,5,10\}$, see Table 5. The graph of the lower bound of $\mathcal { B}^{\delta }_{ Z }(0)$ for the integrated Ornstein-Uhlenbeck process that is $1/\mathbb {E}{\left\{ \epsilon _\delta (Y)\right\} }$ as a function of $\delta \in [0,10]$ is given in Fig. 2. The value of $\mathcal {B}_{ Z }(0)$ constant for the integrated Ornstein-Uhlenbeck process with the same parameters as here was simulated in Dȩbicki et al. (2003) resulting in the value 0.528.

## Further Results and Proofs

Let in the following $X(t),t\in \mathbb {R}^d$ be a max-stable stationary rf with càdlàg sample paths and spectral rf *Z* as in Theorem 2.1 and define $\Theta$ as in (2.9). Define $Y(t)=R \Theta (t),t\in \mathbb {R}^d$ with *R* an 1-Pareto rv (with survival function $1/x, x\geqslant 1$) independent of $\Theta$ and set $M_{Y,\delta }= \sup _{t\in \delta \mathbb {Z}^d} Y(t)$. Note in passing that

$$\begin{aligned} \mathbb {P}{\left\{ M_{Y, \delta } > 1\right\} }=1 \end{aligned}$$

since $M_{Y, \delta } \geqslant R \Theta (0)> 1$ almost surely by the assumption on *R* and by the definition $\mathbb {P}{\left\{ \Theta (0)=1\right\} }=1$.

Recall that $S_\delta =S_\delta (Z)= \int _{\delta \mathbb {Z}^d} Z (t) \lambda _\delta (dt)$. In view of (2.4) we have that $\mathbb {P}{\left\{ S_0>0\right\} }=1$. In the following, for any fixed $\delta \geqslant 0$ (but not simultaneously for two different $\delta$’s) we shall assume that $S_\delta >0$ almost surely, i.e., *Z* is such that $\mathbb {P}{\left\{ \sup _{t\in \delta \mathbb {Z}^d} Z(t)> 0\right\} }=1$. Such a choice of *Z* is possible in view of Hashorva (2021, Lem 7.3).

A functional $F:D \rightarrow [0,\infty ]$ is said to be shift-invariant if $F(f(\cdot -h) )= F(f(\cdot ))$ for all $h\in \mathbb {R}^d$.

We state first two lemmas and proceed with the postponed proofs.

**Table 5:** Estimation of $\mathbb {E}{\left\{ \epsilon _\delta (Y)\right\} }$ and $1/\mathbb {E}{\left\{ \epsilon _\delta (Y)\right\} }$ for $\delta =\{0,0.1,0.2, 0.5,1,2,5,10\}$

$\delta$	0	0.1	0.2	0.5
$\mathbb {E}{\left\{ \epsilon _\delta (Y)\right\} }$	3.234658	3.245584	3.248405	3.268183
$1/\mathbb {E}{\left\{ \epsilon _\delta (Y)\right\} }$	0.3091517	0.308111	0.3078434	0.3059804

$\delta$	1	2	5	10
$\mathbb {E}{\left\{ \epsilon _\delta (Y)\right\} }$	3.339158	3.626068	5.482154	10.05426
$1/\mathbb {E}{\left\{ \epsilon _\delta (Y)\right\} }$	0.2994767	0.2757808	0.1824101	0.09946035

### Lemma 4.1

If $\mathbb {P}{\left\{ S_0 < \infty \right\} }=1$, then $\mathbb {P}{\left\{ S_\delta < \infty \right\} }=1, \delta >0$ and for all $x>0$

4.1 $$\begin{aligned} \mathbb {P}{\left\{ \epsilon _\delta (Y/x)< \infty \right\} }=1, \quad \forall x>0, \forall \delta \geqslant 0. \end{aligned}$$

Moreover $$\mathbb {P}{\left\{ M_{Y, \delta }< \infty \right\} }=1$$.

### Proof of Lemma 4.1

In view of (2.4) $S_0>0$ almost surely. The assumption that $S_0< \infty$ almost surely is in view of Dombry and Kabluchko (2017, Thm 3) equivalent with $Z(t) \rightarrow 0$ almost surely as $\Vert t \Vert \rightarrow \infty$, with $\Vert \cdot \Vert$ some norm on $\mathbb {R}^d$. Hence $S_\delta < \infty$ almost surely follows from Dombry and Kabluchko (2017, Thm 3). By the definition of $\Theta$ and the fact that $\mathbb {P}{\left\{ S_0(Z) \in (0,\infty )\right\} }=1$ we have

4.2 $$\begin{aligned} \begin{aligned} 0&= { \mathbb {E}{\left\{ { Z (0)} \mathbb {I}{\left\{ \limsup _{ \Vert t \Vert \rightarrow \infty } Z(t)>0\right\} } \right\} }} = { \mathbb {E}{\left\{ Z (0)\mathbb {I}{\left\{ \limsup _{ \Vert t \Vert \rightarrow \infty } Z(t) /Z(0)>0\right\} } \right\} }}\\&= \mathbb {E}{\left\{ \mathbb {I}{\left\{ \limsup _{ \Vert t \Vert \rightarrow \infty } \Theta (t) >0\right\} } \right\} } \end{aligned} \end{aligned}$$

implying that $\mathbb {P}{\left\{ \lim _{\Vert t \Vert \rightarrow \infty } \Theta (t) =0\right\} }=1$. Consequently, $\mathbb {P}{\left\{ \lim _{\Vert t \Vert \rightarrow \infty } Y(t) =0\right\} }=1$ and hence the claim follows. $\square$

Below we interpret $\infty \cdot 0$ and 0/0 as 0. The next result is a minor extension of Soulier (2022, Lem 2.7).

### Lemma 4.2

If $\mathbb {P}{\left\{ S_0<\infty \right\} }=1$, then for all measurable shift invariant functional *F* and all $\delta ,x$ non-negative

4.3 $$\begin{aligned} x \mathbb {E}{\left\{ \frac{F(Y/x)}{ \epsilon _\delta (Y)}\mathbb {I}{\left\{ M_{Y,\delta }> {\max }(x,1)\right\} } \right\} } =\mathbb {E}{\left\{ \frac{F(Y)}{ \epsilon _\delta (Y)}\mathbb {I}{\left\{ M_{Y,\delta }> {\max }(1/x,1) \right\} }\right\} }. \end{aligned}$$

### Proof of Lemma 4.2

For all measurable functional $$F: D \rightarrow [0,\infty ]$$ and all $$ x> 0$$

4.4 $$\begin{aligned} x \mathbb {E}{\left\{ F(Y) \mathbbm {I}( { Y(h)}>x ) \right\} }=\mathbb {E}{\left\{ F(x B^h Y) \mathbbm {I}(x { Y(-h)} >1 )\right\} } \end{aligned}$$

is valid for all $h \in \mathbb {R}^d$ with $B^h Y(t)= Y(t-h), h,t\in \mathbb {R}^d$. Note in passing that $B^h Y$ can be substituted by *Y* in the right-hand side of (4.4) if *F* is shift-invariant. The identity (4.4) is shown in Bladt et al. (2022). For the discrete setup it is shown initially in Planinić and Soulier (2018), and Basrak and Planinić (2021) and for case $d=1$ in Soulier (2022).

Next, if $x \in (0,1]$, since $Y(0)=R> 1$ almost surely and by the assumption on the sample paths we have that $\mathbb {P}{\left\{ \epsilon _\delta (Y/x)>0\right\} }=1,$ recall $\mathbb {P}{\left\{ \Theta (0)=1\right\} }=1$. By Lemma 4.1 $\mathbb {P}{\left\{ M_{Y, \delta } \in (1, \infty )\right\} }=1$, hence for all $x> 1$ we have further that $M_{Y, \delta } > x$ implies $\epsilon _\delta (Y/x)>0$. Consequently, in view of (4.1) $\epsilon _\delta (Y/x)/\epsilon _\delta (Y/x)$ is well defined on the event $M_{Y, \delta }> x, x> 1$ and also it is well-defined for any $x\in (0,1]$.

Recall that $\lambda _\delta (dt)$ is the Lebesgue measure on $\mathbb {R}^d$ if $\delta =0$ and the counting measure multiplied by $\delta ^d$ on $\delta \mathbb {Z}^d$ if $\delta >0$. Let us remark that for any shift-invariant functional *F*, the functional

$$\begin{aligned} F^*(Y)=\frac{F(Y/x)\mathbb {I}{\left\{ M_{Y,\delta }> \max (x,1) \right\} }}{\epsilon _\delta (Y)\epsilon _\delta (Y/x)} \end{aligned}$$

is shift-invariant for all $h\in \mathbb {R}^d$ if $\delta =0$ and any shift $h\in \delta \mathbb {Z}^d$ if $\delta >0$. Thus applying the Fubini-Tonelli theorem twice and (4.4) with functional $F^*$ we obtain for all $\delta \geqslant 0,x>0$

$$\begin{aligned}&x\mathbb {E}{\left\{ \frac{F(Y/x)}{\epsilon _\delta (Y)} \mathbb {I}{\left\{ M_{Y,\delta }> \max (x,1) \right\} }\right\} } \\&=x\int _{ \delta \mathbb {Z}^d} \mathbb {E}{\left\{ \frac{F(Y/x)\mathbb {I}{\left\{ M_{Y,\delta }> \max (x,1) \right\} }}{\epsilon _\delta (Y)\epsilon _\delta (Y/x) } \mathbb {I}{\left\{ Y(h)> x\right\} }\right\} } \lambda _\delta (dh)\\&= \int _{ \delta \mathbb {Z}^d} \mathbb {E}{\left\{ \frac{F(Y)\mathbb {I}{\left\{ M_{Y,\delta }> \max (1/x,1) \right\} }}{\epsilon _\delta (xY)\epsilon _\delta (Y) } \mathbb {I}{\left\{ xY(-h)> 1\right\} }\right\} } \lambda _\delta (dh)\\&= \mathbb {E}{\left\{ \frac{F(Y)\mathbb {I}{\left\{ M_{Y,\delta }> \max (1/x,1) \right\} }}{\epsilon _\delta (xY)\epsilon _\delta (Y) } \int _{ \delta \mathbb {Z}^d}\mathbb {I}{\left\{ xY(h)> 1\right\} } \lambda _\delta (dh)\right\} } \\&=\mathbb {E}{\left\{ \frac{F(Y)}{ \epsilon _\delta (Y)}\mathbb {I}{\left\{ M_{Y,\delta }> \max (1/x,1) \right\} }\right\} }, \end{aligned}$$

hence the proof follows. $\square$

### Proof of Theorem 2.1

Let $\delta \geqslant 0$ be fixed and consider for simplicity $d=1$. By the assumption we have $\mathbb {E}{\left\{ \sup _{t\in [0,T]} Z (t)\right\} }\!<\! \infty$ for all $T\!>\!0$. Since we assume that $\mathbb {P}{\left\{ \sup _{t \in \mathbb {R}} Z(t)\!>\!0\right\} }\!=\!1$, then $\mathbb {P}{\left\{ S_0\!>\!0\right\} }=1$. Using the assumption we have $\mathbb {P}{\left\{ S_\eta < \infty \right\} }>0$ for all $\eta \geqslant 0$ and thus by (2.3) we obtain

$$\begin{aligned} \infty> & {} \mathbb {E}{\left\{ \sup _{t\in [0,{2}] } Z (t) \frac{S_0}{S_0}\right\} } = \int _{\mathbb {R}}\mathbb {E}{\left\{ Z (h) \sup _{t\in [0,2] } Z (t) /S_0\right\} } \lambda (dh)\\= & {} \int _{\mathbb {R}} \mathbb {E}{\left\{ Z (0) \sup _{t\in [-h,2-h] } Z (t) /S_0\right\} } \lambda (dh)\\= & {} {\sum _{i\in \mathbb {Z}} \int _i^{i+1}\mathbb {E}{\left\{ Z (0) \sup _{t\in [-h, 2-h] } Z(t) /S_0\right\} } \lambda (dh)}\\&{\ge }&\sum _{i\in \mathbb {Z}} \mathbb {E}{\left\{ Z(0) \sup _{t\in [-i,{1}-i] } Z(t) /S_0\right\} } \\\geqslant & {} \mathbb {E}{\left\{ Z(0) \sup _{t\in \mathbb {R}} Z(t) /S_0 \right\} }. \end{aligned}$$

Since also for any $M>0$ and $\eta >0$

$$\begin{aligned} 0< \mathbb {E}{\left\{ \sup _{t\in [0,{M}] } Z (t) \frac{S_\eta }{S_\eta }\right\} } < \infty \end{aligned}$$

we conclude as above that for all $\eta \geqslant 0$

4.5 $$\begin{aligned} \mathbb {E}{\left\{ Z(0) \sup _{t\in \mathbb {R}} Z (t) /S_\eta \right\} }< \infty , \quad \int _{\eta \mathbb {Z}} \mathbb {E}{\left\{ Z (0) \sup _ {h \leqslant t\leqslant h +1} Z (t) /S_\eta \right\} }\lambda _\eta (dh) < \infty . \end{aligned}$$

Next, for any $x\geqslant 0$ and $\eta \geqslant 0$

$$\begin{aligned}{} & {} T^{-1} \int _0^\infty \mathbb {P}{\left\{ \int _{[0,T] \cap \eta \mathbb {Z}} \mathbbm {I}( Z (t)> s) \lambda _\eta (dt)> x, S_\eta =\infty \right\} } ds\\{} & {} \leqslant T^{-1} \int _0^\infty \mathbb {P}{\left\{ \int _{[0,T] \cap \eta \mathbb {Z}} \mathbbm {I}( Z (t)> s) \lambda _\eta (dt)> 0, S_\eta =\infty \right\} } ds \\{} & {} = T^{-1} \int _0^\infty \mathbb {P}{\left\{ \sup _{t\in [0,T] \cap \eta \mathbb {Z}} Z (t) > s, S_\eta =\infty \right\} } ds \\{} & {} = T^{-1} \mathbb {E}{\left\{ \sup _{t\in [0,T] \cap \eta \mathbb {Z}} Z (t), S_\eta =\infty \right\} } \\{} & {} \rightarrow 0, \quad T \rightarrow \infty , \end{aligned}$$

where the last claim follows from Dȩbicki et al. (2022, Cor 2.1). We shall assume that $S_\delta >0$ almost surely (this is possible as mentioned at the beginning of this section). For notational simplicity we consider next $\delta =0$. For any $M>0, T> 2M$ by the Fubini-Tonelli theorem and (2.3)

$$\begin{aligned}{} & {} \int _0^\infty \mathbb {P}{\left\{ \int _{[0,T] } \mathbbm {I}( Z (t)> s) \lambda (dt)> x \right\} } ds \\{} & {} = \int _{\mathbb {R}} \mathbb {E}{\left\{ \frac{ Z (h)}{S_{{0}}} \int _0^\infty \mathbb {I}{\left\{ \int _{[0,T] } \mathbbm {I}( Z (t)> s) \lambda (dt)> x \right\} } ds \right\} } dh \\{} & {} = \int _{\mathbb {R}} \mathbb {E}{\left\{ \frac{ Z (0)}{{S_{{0}}}} \int _0^\infty \mathbb {I}{\left\{ \int _{[0,T]} \mathbbm {I}( Z (t-h)> s) \lambda (dt)> x \right\} }ds\right\} } dh\\{} & {} = \int _{\mathbb {R}} \int _0^\infty \mathbb {E}{\left\{ \frac{ Z (0)}{S_{{0}}} \mathbb {I}{\left\{ \int _{h}^{ T+h} \mathbbm {I}( Z (t)> s) \lambda (dt)> x \right\} }\right\} } dsdh\\{} & {} = \int _{-M-T}^{-M}\int _0^\infty \mathbb {E}{\left\{ \frac{ Z (0)}{S_{{0}}} \mathbb {I}{\left\{ \int _h^{ T+h} \mathbbm {I}( Z (t)> s) \lambda (dt)> x \right\} }\right\} } dsdh\\{} & {} \quad + \int _{h<-M-T \,\text {or}\, h>-M}\int _0^\infty \mathbb {E}{\left\{ \frac{ Z (0)}{S_{{0}}} \mathbb {I}{\left\{ \int _h^{ T+h} \mathbbm {I}( Z (t)> s) \lambda (dt) > x \right\} }\right\} } dsdh\\{} & {} =:I_{M,T}+ J_{M,T}. \end{aligned}$$

Thus we obtain

$$\begin{aligned} \frac{I_{M,T}}{T}= & {} \int _{-M/T-1}^{-M/T} \mathbb {E}{\left\{ \frac{ Z (0)}{S_{{0}}} \int _0^\infty \mathbbm {I} \Bigl ( \int _{Th}^{T(1+h)} \mathbbm {I}( Z (t)> s) \lambda (dt)> x \Bigr ) ds \right\} } dh\\= & {} \int _{-1}^{0} \mathbb {E}{\left\{ \frac{ Z (0)}{S_{{0}}} \int _0^\infty \mathbbm {I} \Bigl ( \int _{Th-M}^{T(1+h)-M} \mathbbm {I}( Z (t)> s) \lambda (dt)> x \Bigr ) ds\right\} } dh\\\rightarrow & {} \int _{-1}^{0} \mathbb {E}{\left\{ \frac{ Z (0)}{S_{{0}}} \int _0^\infty \mathbbm {I} \Bigl ( \int _{\delta \mathbb {Z}} \mathbbm {I}( Z (t)> s) \lambda (dt)> x \Bigr ) ds \right\} } dy, \quad T\rightarrow \infty \\= & {} \mathbb {E}{\left\{ \frac{ Z (0)}{S_{{0}}} \int _0^\infty \mathbbm {I} \Bigl ( \int _{\delta \mathbb {Z}} \mathbbm {I}( Z (t)> s ) \lambda _\delta (dt)> x \Bigr ) ds \right\} }\\\leqslant & {} \mathbb {E}{\left\{ \frac{ Z (0)}{S_{{0}}} \int _0^\infty \mathbbm {I} \Bigl ( \int _{\mathbb {R}} \mathbbm {I}( Z (t)> s ) \lambda (dt) > 0 \Bigr ) ds\right\} }\\= & {} \mathbb {E}{\left\{ \frac{ Z (0)}{S_{{0}}} \sup _{t\in \mathbb {R}} Z (t)\right\} } = {\mathcal {H}_{ Z }^{{0}} }< \infty , \end{aligned}$$

where ${\mathcal {H}^{{0}}_{ Z }}$ is the Pickands constants, see Dȩbicki et al. (2022, Prop 2.1) for the last formula.

Let us consider the second term

$$\begin{aligned} \frac{J_{M,T}}{T}= & {} \int _{(-\infty , -1)\cup (0,\infty )} \mathbb {E}{\left\{ \frac{ Z (0)}{S_0} \int _0^\infty \mathbbm {I} \Bigl ( \int _{Th-M}^{T(1+h)-M} \mathbbm {I}( Z (t)> s) \lambda (dt)> x \Bigr ) ds \right\} } dh\\= & {} \int _{-\infty }^{-1} \mathbb {E}{\left\{ \frac{ Z (0)}{S_0} \int _0^\infty \mathbbm {I} \Bigl ( \int _{Th-M}^{T(1+h)-M} \mathbbm {I}( Z (t)> s) \lambda (dt)> x \Bigr ) ds \right\} } dh\\{} & {} + \int _0^\infty \mathbb {E}{\left\{ \frac{ Z (0)}{S_0} \int _0^\infty \mathbbm {I} \Bigl ( \int _{Th-M}^{T(1+h)-M} \mathbbm {I}( Z (t)> s) \lambda (dt) > x \Bigr ) ds \right\} } dh\\=: & {} K_{M,T}+L_{M,T}. \end{aligned}$$

Further, assuming for simplicity that *T* is a positive integer we get

$$\begin{aligned} K_{M,T}\le & {} \int _{-\infty }^{-1} \mathbb {E}{\left\{ \frac{ Z (0)}{S_0} \int _0^\infty \mathbbm {I} \Bigl ( \int _{Th-M}^{T(1+h)-M} \mathbbm {I}( Z (v)> s) \lambda (dv) > 0 \Bigr ) ds \right\} } dh\\= & {} \int _{-\infty }^{-1} \mathbb {E}{\left\{ \frac{ Z (0)}{S_0} \sup _{t\in [Th-M,\, T(1+h)-M]} Z (t)\right\} } dh\\= & {} \frac{1}{T}\int _{-\infty }^{-T-M} \mathbb {E}{\left\{ \frac{ Z (0)}{S_0} \sup _{t\in [h,\, h+T]} Z (t)\right\} } dh\\\le & {} \frac{1}{T}\sum _{i=1}^{T-1}\int _{-\infty }^{-T-M} \mathbb {E}{\left\{ \frac{ Z (0)}{S_0} \sup _{t\in [h+i,\, h+i+1]} Z (t)\right\} } dh\\= & {} \frac{1}{T}\sum _{i=1}^{T-1}\int _{-\infty }^{-T-M+i} \mathbb {E}{\left\{ \frac{ Z (0)}{S_0} \sup _{t\in [h,\, h+1]} Z (t)\right\} } dh\\\le & {} \frac{1}{T}\sum _{i=1}^{T-1}\int _{-\infty }^{-M} \mathbb {E}{\left\{ \frac{ Z (0)}{S_0} \sup _{t\in [h,\, h+1]} Z (t)\right\} } dh\\= & {} \int _{-\infty }^{-M} \mathbb {E}{\left\{ \frac{ Z (0)}{S_0} \sup _{t\in [h,\, h+1]} Z (t)\right\} } dh \rightarrow 0, \quad M \rightarrow \infty , \end{aligned}$$

where the last convergence follows from (4.5). The same way we show that $L_{M,T}\rightarrow 0$ as $M\rightarrow \infty$ establishing the proof.

We prove next the second claim. In view of Dȩbicki et al. (2022, proof of Prop 2.1) almost surely for all $\delta ,\eta \in [0,\infty )$

4.6 $$\begin{aligned} \frac{ 1 }{S_\eta (\Theta )} =\frac{ 1 }{S_\eta (\Theta )} \frac{ S_\delta (\Theta )}{S_\delta (\Theta )}\Theta (0), \quad \{S_\eta (\Theta )<\infty \} = \{S_\delta (\Theta ) <\infty \}. \end{aligned}$$

Consequently, for any $\delta ,\eta , x$ non-negative

$$\begin{aligned}{} & {} { B_{\delta , \eta }(x)}\\{} & {} := \int _0^\infty \mathbb {E}{\left\{ \frac{ Z (0)}{ S_\eta } \mathbb {I}{\left\{ S_\eta< \infty \right\} }\mathbbm {I} \Bigl ( \int _{\delta \mathbb {Z}} \mathbbm {I}( Z (t)> s) \lambda _\delta (dt)> x \Bigr ) \right\} }ds\\{} & {} = \int _0^\infty \mathbb {E}{\left\{ \frac{1 }{ S_\eta (\Theta )} \mathbb {I}{\left\{ S_\eta (\Theta )< \infty \right\} } \mathbbm {I} \Bigl ( \int _{ \delta \mathbb {Z}} \mathbbm {I}( \Theta (t)> s) \lambda _\delta (dt)> x \Bigr ) \right\} }ds\\{} & {} = \int _0^\infty \mathbb {E}{\left\{ \frac{1 }{ S_\eta (\Theta )} \frac{S_\delta (\Theta )}{S_\delta (\Theta )} \mathbb {I}{\left\{ S_\delta (\Theta )< \infty \right\} } \mathbbm {I} \Bigl ( \int _{ \delta \mathbb {Z}} \mathbbm {I}( \Theta (t)> s) \lambda _\delta (dt) > x \Bigr ) \right\} }ds. \end{aligned}$$

We proceed next with the case $\delta =0$, the other case follows with the same argument where it is important that $\eta =k\delta$ for the shift transformation. Taking $\delta =0, \eta >0$ we have

$$\begin{aligned}{} & {} { B_{0, \eta }(x)}\\{} & {} = \int _{\mathbb {R}} \int _0^\infty \mathbb {E}{\left\{ \frac{1 }{ S_\eta (\Theta )} \frac{\Theta (r)}{S_0(\Theta )} \mathbb {I}{\left\{ S_0(\Theta )< \infty \right\} } \mathbbm {I} \Bigl ( \int _{ \mathbb {R}} \mathbbm {I}( \Theta (t)> s) \lambda (dt)> x \Bigr ) \right\} }ds\lambda (dr)\\{} & {} = \sum _{v\in \eta \mathbb {Z}} \int _{ r+v\in [0,\eta ]} \int _0^\infty \mathbb {E}{\left\{ \frac{ Z (0) }{ S_\eta (Z)} \frac{ Z (r)}{S_0(Z)} \mathbb {I}{\left\{ S_0(Z)< \infty \right\} } \mathbbm {I} \Bigl ( \int _{ \mathbb {R}} \mathbbm {I}( Z (t)> s) \lambda (dt)> x \Bigr ) \right\} }ds\lambda (dr)\\{} & {} = \int _0^\infty \mathbb {E}{\left\{ \sum _{v\in \eta \mathbb {Z}} \int _{ r +v\in [0,\eta ]}\frac{ Z (0) }{ S_\eta (Z)} \frac{ Z (r)}{S_0(Z)} \mathbb {I}{\left\{ S_0(Z)< \infty \right\} } \mathbbm {I} \Bigl ( \int _{ \delta \mathbb {Z}} \mathbbm {I}( Z (t)> s) \lambda (dt)> x \Bigr ) \right\} }\lambda (dr) ds\\{} & {} = \int _0^\infty \mathbb {E}{\left\{ \sum _{v\in \eta \mathbb {Z}} \int _{ r\in [0,\eta ]}\frac{ Z (0) }{ S_\eta (Z)} \frac{ Z (r-v)}{S_0(Z)} \mathbb {I}{\left\{ S_0(Z)< \infty \right\} } \mathbbm {I} \Bigl ( \int _{ \mathbb {R}} \mathbbm {I}( Z (t)> s) \lambda (dt)> x \Bigr ) \right\} }\lambda (dr) ds\\{} & {} = \int _0^\infty \mathbb {E}{\left\{ \int _{ r\in [0,\eta ]} \frac{1}{\eta } \sum _{v\in \eta \mathbb {Z}} \frac{ \eta Z (v-r) }{ S_\eta ({B^r}Z)} \lambda (dr) \frac{ Z (0)}{S_0(Z)} \mathbb {I}{\left\{ S_0(Z)< \infty \right\} } \mathbbm {I} \Bigl ( \int _{ \mathbb {R}} \mathbbm {I}( Z (t)> s) \lambda (dt)> x \Bigr ) \right\} } ds\\{} & {} = \int _0^\infty \mathbb {E}{\left\{ \frac{ Z (0)}{S_0(Z)} \mathbbm {I} \Bigl ( \int _{ \mathbb {R}} \mathbbm {I}( Z (t)> s) \lambda (dt) > x \Bigr ) \right\} }ds, \end{aligned}$$

where we used (2.3) with $h=r-v$ to obtain the second last equality above and (2.6) to get the last equality, hence the proof follows. $\square$

### Proof of Corollary 2.3

Given $x\geqslant 0$ consider the representation (2.5)

$$\begin{aligned} \mathcal {B}_{ Z }^0(x) = \int _0^\infty \mathbb {E}{\left\{ \frac{ Z (0)}{ S_0} \mathbbm {I} \Bigl ( \int _{\mathbb {R}^d} \mathbbm {I}( Z (t)> s) \lambda (dt) > x \Bigr ) \right\} }ds. \end{aligned}$$

By the monotonicity with respect to variable *x* of the function

4.7 $$\begin{aligned} \mathbb {E}{\left\{ \frac{ Z (0)}{ S_{0}} \mathbbm {I} \Bigl ( \int _{\mathbb {R}^d} \mathbbm {I}( Z (t)> s) \lambda (dt) > x \Bigr ) \right\} } \end{aligned}$$

in order to show the continuity of $\mathcal {B}_{ Z }^0(x)$ it suffices to prove that

4.8 $$\begin{aligned} \mathbb {E}{\left\{ \frac{ Z (0)}{ S_{0}} \mathbbm {I} \Bigl ( \int _{\mathbb {R}^d} \mathbbm {I}( Z (t) > s) \lambda (dt) = x \Bigr ) \right\} }=0 \end{aligned}$$

for almost all $s>0$. Let us define the following measurable sets

$$\begin{aligned} A_s=\mathbbm {I} \Bigl ( \int _{\mathbb {R}^d} \mathbbm {I}( Z (t)>s) \lambda (dt) = x \Bigr )\,. \end{aligned}$$

Since *Z* has almost surely continuous trajectories we have $A_s\cap A_{s'}=\emptyset$ if $0<s<s'$ and $x>0$. Thus there are countably many $s>0$ such that $\mathbb {P}{\left\{ A_s\right\} }>0$ because if there were not countably many ones we would find countably many disjoint $A_s$ such that $\sum \mathbb {P}{\left\{ A_s\right\} }=\infty$. Thus we get (4.8) for almost all $s>0$. The continuity at $x=0$ follows from the right continuity of (4.7). $\square$

### Proof of Lemma 2.4

Item (i): In view of (2.5) and substituting $\Theta (t)=Q_\delta (t)/ S_\delta (\Theta )$ to (2.10) we get

$$\begin{aligned} \mathcal {B}_{ Z }^\delta (x)= \int _0^\infty \mathbb {E}{\left\{ \frac{1}{S_\delta (Q_\delta )}\mathbbm {I} \Bigl ( \int _{\delta \mathbb {Z}^d} \mathbbm {I}( Q_\delta (t)> s) \lambda _\delta (dt) > x \Bigr ) \right\} }ds. \end{aligned}$$

Since $S_\delta (Q_\delta )=1$ the claim follows.

Item (ii): If $\mathbb {P}{\left\{ S_0< \infty \right\} } > 0$ we can define $V(t)= Z(t) |{ S_0 < \infty }$ and set (recall $S_0=S_0(Z), \mathbb {E}{\left\{ Z (0)\right\} }=1$)

$$\begin{aligned} b= \mathbb {E}{\left\{ Z (0)\mathbb {I}{\left\{ S_0< \infty \right\} }\right\} }= \mathbb {P}{\left\{ S_0(\Theta )< \infty \right\} }>0. \end{aligned}$$

For this choice of *b* by (2.3) we have

$$\begin{aligned} \mathbb {E}{\left\{ V (t)\right\} } = \frac{\mathbb {E}{\left\{ Z(t) \mathbb {I}{\left\{ S_0< \infty \right\} } \right\} }}{ \mathbb {P}{\left\{ S_0(\Theta )< \infty \right\} }} =\frac{\mathbb {E}{\left\{ Z(0) \mathbb {I}{\left\{ S_0< \infty \right\} } \right\} }}{ \mathbb {P}{\left\{ S_0(\Theta )< \infty \right\} }}= 1 \end{aligned}$$

for all $t\in \mathbb {R}$. Clearly, $\mathbb {P}{\left\{ \sup _{t\in \mathbb {R}^d} V(t)>0\right\} } =1$. In view of Dombry and Kabluchko (2017) *V* is the spectral rf of a stationary max-stable rf $X_*$ with càdlàg sample paths and moreover $S_0(V)=\int _{\mathbb {R}^d} V (t) \lambda (dt)<\infty$ almost surely. In view of Dȩbicki et al. (2022, proof of Prop. 2.1) we have that

$$\begin{aligned} \{S_\delta (\Theta )<\infty \}= \{S_0(\Theta )<\infty \} \end{aligned}$$

almost surely for all $\delta >0$. Consequently, we obtain for all $\delta >0$

$$\begin{aligned} \mathcal { B}_{ Z }^ \delta (x)= & {} \int _0^\infty \mathbb {E}{\left\{ \frac{ Z (0)}{ S_\delta } \mathbbm {I} \Bigl ( \int _{\delta \mathbb {Z}^d} \mathbbm {I}( Z (t)> s) \lambda _\delta (dt)> x \Bigr ) \mathbb {I}{\left\{ S_\delta< \infty \right\} }\right\} }ds\\= & {} \int _0^\infty \mathbb {E}{\left\{ \frac{ 1}{ S_\delta (\Theta ) } \mathbbm {I} \Bigl ( \int _{\delta \mathbb {Z}^d} \mathbbm {I}( \Theta (t)> s) \lambda _\delta (dt)> x \Bigr ) \mathbb {I}{\left\{ S_\delta (\Theta )< \infty , S_0(\Theta )< \infty \right\} } \right\} }ds\\= & {} \int _0^\infty \mathbb {E}{\left\{ \frac{ Z (0)}{ S_\delta (Z) } \mathbbm {I} \Bigl ( \int _{\delta \mathbb {Z}^d} \mathbbm {I}( Z (t)> s) \lambda _\delta (dt)> x \Bigr ) \mathbb {I}{\left\{ S_0(Z)< \infty \right\} } \right\} } ds\\= & {} b \int _0^\infty \mathbb {E}{\left\{ \frac{ V(0)}{ S_\delta (V) } \mathbbm {I} \Bigl ( \int _{\delta \mathbb {Z}^d} \mathbbm {I}( V (t)> s) \lambda _\delta (dt) > x \Bigr ) \right\} }ds\\= & {} b\mathcal { B}_{ V}^ \delta (x) < \infty \end{aligned}$$

establishing the proof. $\square$

### Proof of Theorem 2.5

Assume first that $\mathbb {P}{\left\{ S_0< \infty \right\} }=1$. In view of (4.1) we have that $\epsilon _\delta < \infty$ almost surely, hence as in Kulik and Soulier (2020), and Soulier (2022) where $d=1$ is considered it follows that (2.12) holds with

$$\begin{aligned} {\bar{Q}_\delta (t)}= c \frac{ Y(t)}{ \epsilon _\delta (Y) M_{Y,\delta }} , \quad t\in \mathbb {R}, \end{aligned}$$

with $c=1$ if $\delta =0$ and $c=\delta ^d$ otherwise. Set below $Q_\delta = \bar{Q}/c$ and for simplicity omit the subscript below writing simply $M_Y$ instead of $M_{Y,\delta }$. Since $Y(t)/M_Y \leqslant 1$ almost surely for all $t\in \delta \mathbb {Z}^d$ and $\mathbb {P}{\left\{ M_Y\in (1,\infty )\right\} }=1$, in view of Lemma 2.4 we have using further the Fubini-Tonelli theorem and Lemma 4.2

$$\begin{aligned} \mathcal { B}_{ Z }^\delta (x)= & {} \int _0^\infty \mathbb {E}{\left\{ \mathbbm {I} \Bigl ( \int _{\delta \mathbb {Z}^d} \mathbbm {I}( {Q} _\delta (v)> s) \lambda _\delta (dv)> x \Bigr ) \right\} }ds \\= & {} \int _0^\infty \mathbb {E}{\left\{ \frac{1}{\epsilon _\delta (Y) } \frac{1}{M_Y } \mathbb {I}{\left\{ \epsilon _\delta (Y/s)> x\right\} }\right\} } ds\\= & {} \int _0^\infty \mathbb {E}{\left\{ \frac{1}{\epsilon _\delta (Y) } \mathbb {I}{\left\{ M_Y> s\right\} } \frac{1}{M_Y } \mathbb {I}{\left\{ \epsilon _\delta (Y/s)> x\right\} }\right\} } ds\\=: & {} \int _0^\infty \mathbb {E}{\left\{ \frac{1}{ {s}\epsilon _\delta (Y) } \mathbb {I}{\left\{ M_Y> s\right\} } F(Y/s) \right\} } ds\\= & {} \int _0^\infty \frac{1}{s^2} \mathbb {E}{\left\{ \frac{ \mathbb {I}{\left\{ \epsilon _\delta (Y)> x\right\} } }{\epsilon _\delta (Y ) M_Y } \mathbb {I}{\left\{ M_Y> \max (1/s,1)\right\} } \right\} } ds\\= & {} \mathbb {E}{\left\{ \frac{ \mathbb {I}{\left\{ \epsilon _\delta (Y)> x\right\} }}{\epsilon _\delta (Y ) M_Y } \int _0^\infty \frac{1}{s^2} \mathbb {I}{\left\{ M_Y> \max (1/s,1)\right\} } ds\right\} }\\= & {} \mathbb {E}{\left\{ \frac{ \mathbb {I}{\left\{ \epsilon _\delta (Y) > x\right\} }}{\epsilon _\delta (Y )} \right\} }. \end{aligned}$$

The last equality follows from (recall $M_Y\in (1,\infty )$ almost surely)

$$\begin{aligned} \int _0^\infty \frac{1}{s^2} \mathbb {I}{\left\{ M_Y> \max (1/s,1)\right\} } ds= \int _1^\infty \frac{1}{s^2} ds+\int _0^1 \frac{1}{s^2} \mathbb {I}{\left\{ M_Y > 1/s\right\} } ds=M_Y. \end{aligned}$$

In view of (4.1) for all *x* non-negative such that $\mathbb {P}{\left\{ \epsilon _\delta (Y)>x\right\} }>0$ we have that $\mathcal { B}_{ Z }^\delta (x) \in (0,\infty )$, hence the proof follows.

Assume now that $\mathbb {P}{\left\{ S_0< \infty \right\} }\in (0,1)$. In view of Lemma 2.4 we have

$$\begin{aligned} \mathcal { B}_{ Z }^ \delta (x)= b\mathcal { B}_{ V}^ \delta (x), \end{aligned}$$

with $V(t)= Z(t)|S_0 < \infty$, which is well-defined since $\mathbb {P}{\left\{ S_0<\infty \right\} }>0$ by the assumption. Since $S_0(V)< \infty$ almost surely and $Y_*(t) =Y(t) |S_0(\Theta )< \infty ,t\in \mathbb {R}$ by the proof above

$$\begin{aligned} \mathcal { B}_{ Z }^ \delta (x)= & {} \mathbb {P}{\left\{ S_0< \infty \right\} }\mathbb {E}{\left\{ \frac{ \mathbb {I}{\left\{ \epsilon _\delta (Y_*)> x\right\} }}{\epsilon _\delta (Y_* )}\right\} } \\= & {} \mathbb {E}{\left\{ \frac{ \mathbb {I}{\left\{ \epsilon _\delta (R \Theta ) > x\right\} }}{\epsilon _\delta (R \Theta )} \mathbb {I}{\left\{ S_0(\Theta )< \infty \right\} }\right\} }. \end{aligned}$$

In view of Soulier (2022, Lem 2.5, Cor 2.9) and Hashorva (2021, Thm 3.8) and the above

4.9 $$\begin{aligned} H_{ Z }^\delta =\mathcal { B}_{ Z }^ \delta (0)=\mathbb {E}{\left\{ \frac{ 1}{\epsilon _\delta (R \Theta )}\right\} }=\mathbb {E}{\left\{ \frac{ 1}{\epsilon _\delta (R \Theta )} \mathbb {I}{\left\{ S_0(\Theta )< \infty \right\} }\right\} } \end{aligned}$$

and thus $\epsilon _\delta (R \Theta )< \infty$ implies $S_0< \infty$ almost surely. Hence the proof is complete. $\square$

### Proof of Corollary 2.6

In view of (), the representation (2.15) and the finiteness of $\mathcal {B}_Z^0(x)$ for all $x\geqslant 0$, the monotone convergence theorem yields for all $x_0\geqslant 0$

$$\begin{aligned} \lim _{x \downarrow x_0} \mathbb {E}{\left\{ \frac{\mathbb {I}{\left\{ x_0 \leqslant \epsilon _0 (Y )<x\right\} }}{\epsilon _0 (Y )} \right\} } =\mathbb {E}{\left\{ \frac{\mathbb {I}{\left\{ \epsilon _0 (Y )=x_0\right\} }}{\epsilon _0 (Y )} \right\} }=0 \end{aligned}$$

consequently, since by our assumption Lemma 4.1 implies $\mathbb {P}{\left\{ \epsilon _0 (Y ) \in (0,\infty )\right\} }=1$, then

$$\begin{aligned} \mathbb {P}{\left\{ \epsilon _0 (Y )=x_0 \right\} } = \mathbb {E}{\left\{ \mathbb {I}{\left\{ \epsilon _0 (Y )=x_0\right\} }\right\} }=0 \end{aligned}$$

follows establishing the claim. $\square$

### Proof of Proposition 2.7

In order to prove (2.16) note first that for any non-negative rv *U* with df *G* and $x\geqslant 0$ such that $\mathbb {P}{\left\{ U>x\right\} }>0$

$$\begin{aligned} \frac{1}{\mathbb {P}{\left\{ U>x\right\} }}\int _x^\infty \frac{1}{y}dG(y)\geqslant & {} \frac{\mathbb {P}{\left\{ U>x\right\} }}{\int _x^\infty ydG(y)}\geqslant \frac{\mathbb {P}{\left\{ U>x\right\} }}{\mathbb {E}{\left\{ U\right\} }}. \end{aligned}$$

Consequently, we obtain for all $x>0$

$$\begin{aligned} \mathcal { B}_{ Z }^\delta (x)\geqslant & {} \frac{\mathbb {P}{\left\{ \epsilon _\delta (Y)>x\right\} }^2}{\mathbb {E}{\left\{ \epsilon _\delta (Y)\mathbbm {I} \{\epsilon _\delta (Y)>x\}\right\} }} \geqslant \frac{\mathbb {P}{\left\{ \epsilon _\delta (Y)>x\right\} }^2}{\mathbb {E}{\left\{ \epsilon _\delta (Y)\right\} }} \end{aligned}$$

establishing the proof of the lower bound (2.16). The proof of the upper bound follows from the fact that

$$\begin{aligned} \mathcal { B}_{ Z }^\delta (x) = \int _{x}^\infty \frac{1}{y}dF(y) \leqslant \frac{1}{x}\int _{x}^\infty dF(y)=x^{-1}{\mathbb {P}{\left\{ \epsilon _\delta (Y)\geqslant x\right\} }}, \end{aligned}$$

where *F* is the distribution of $\epsilon _\delta (Y)$. This completes the proof. $\square$

### Proof of Proposition 3.1

Since $\mathcal { B}_{ Z }^\delta (0)$ is the generalised Pickands constant $\mathcal {H}_{ Z }^\delta$, then the claim follows for $x=0$ from Dȩbicki et al. (2022). In view of (2.14) we can assume without loss of generality that $\mathbb {P}{\left\{ S_0 < \infty \right\} }=1$. Under this assumption, from the proof of Lemma 4.1 we have that $Y(t)\rightarrow 0$ almost surely as $\Vert t \Vert \rightarrow \infty$. Hence for some *M* sufficiently large $Y(t) < 1$ almost surely for all *t* such that $\Vert t \Vert > M$. Consequently, for all $\delta \geqslant 0$

$$\begin{aligned} \epsilon _\delta (Y) = \int _{\delta Z ^d \cap [-M, M]^d} \mathbb {I}{\left\{ Y(t)> 1\right\} } \lambda _\delta (dt). \end{aligned}$$

Moreover, $\epsilon _\delta (Y)< \infty$ almost surely for all $\delta \geqslant 0$ implying $\epsilon _\delta (Y) \rightarrow \epsilon _0(Y)$ almost surely as $\delta \downarrow 0$. In view of Soulier (2022, Lem. 2.5, Cor. 2.9) and Hashorva (2021, Thm 3.8) for all $\delta \geqslant 0$

$$\begin{aligned} \mathcal {H}_{ Z }^\delta =\mathbb {E}{\left\{ 1/\epsilon _\delta (Y) \right\} }. \end{aligned}$$

Applying Dȩbicki et al. (2022, Thm 2) and (4.9) yields

$$\begin{aligned} \mathbb {E}{\left\{ 1/\epsilon _\delta (Y) \right\} } = \mathcal {H}_{ Z }^{\delta } \rightarrow \mathcal {H}_{ Z }^0= \mathbb {E}{\left\{ 1/\epsilon _0(Y) \right\} }, \quad \delta \downarrow 0. \end{aligned}$$

Hence $1/\epsilon _{\delta }(Y), \delta >0$ is uniformly integrable and hence

$$\begin{aligned} \mathcal {B}_{ Z }^\delta (x)= \mathbb {E}{\left\{ \frac{ \mathbb {I}{\left\{ \epsilon _\delta (Y) > x \right\} }}{\epsilon _\delta (Y) }\right\} } \rightarrow \mathcal {B}_{ Z }^0(x), \quad \delta \downarrow 0 \end{aligned}$$

establishing the proof. $\square$

### Proof of Theorem 3.2

Suppose that $V(t), t\in \mathbb {R}^d$ is a centered Gaussian field with stationary increments and variance function $\sigma ^2_V(\cdot )$ that satisfies **A1-A2**. Then, by stationarity of increments $\sigma ^2_V(\cdot )$ is negative definite, which combined with Schoenberg’s theorem, implies that for each $u>0$

$$\begin{aligned} R_u(s,t):=\exp \left( -\frac{1}{2u^2}\sigma _V^2(s-t)\right) , \ s,t\in \mathbb {R}^d \end{aligned}$$

is positive definite, and thus a valid covariance function of some centered stationary Gaussian rf $X_u(t), t\in \mathbb {R}^d$, where $s-t$ is meant component-wise. The proof of Theorem 3.2 is based on the analysis of the asymptotics of sojourn time of $X_u(t)$. Since the idea of the proof is the same for continuous and discrete scenario, in order to simplify notation, we consider next only the case $\delta =0$.

Before we proceed to the proof of Theorem 3.2, we need the following lemmas, where $Z(t)=\exp \left( V(t)-\frac{\sigma ^2_V(t)}{2}\right)$ is as in Remark 2.2, Item (iii).

#### Lemma 4.3

For all $T>0$ and $x\geqslant 0$

(i) $$\begin{aligned} \lim _{u\rightarrow \infty }\frac{\mathbb {P}{\left\{ \int _{[0,T]^d } \mathbbm {I}(X_u(t)>u) dt>x \right\} }}{\Psi (u)}=\mathcal {B}_Z([0,T]^d,x). \end{aligned}$$
(ii) For all $x\geqslant 0$
  $$\begin{aligned}&\lim _{u\rightarrow \infty }\frac{\mathbb {P}{\left\{ \int _{[0,\ln (u)]^d } \mathbbm {I}(X_u(t)>u) dt>x \right\} }}{(\ln (u))^d\Psi (u)}=\mathcal {B}_Z(x),\\&\lim _{T\rightarrow \infty }\frac{\mathcal {B}_Z([0,T]^d,x)}{T^d}=\mathcal {B}_Z(x)\in (0,\infty ). \end{aligned}$$

#### Proof of Lemma 4.3

Item (i) follows straightforwardly from Dȩbicki et al. (2023, Lem. 4.1). The proof of Item (i) follows by the application of the double sum technique applied to the sojourn functional, as demonstrated e.g., in Dȩbicki et al. (2023, Prop. 3.1). The claim in Item (ii) follows by the same argument as its counterpart in Dȩbicki et al. (2023, Lem. 4.2). $\square$

The following lemma is a slight modification of Piterbarg (1996, Lem 6.3) to the family $X_u,\ u>0$. Let ${\textbf {i}}=(i_1,...,i_d)$, with $i_1,...,i_d\in \{0,1,2,...\}$, $\mathcal {R}_{{\textbf {i}}}:=\prod _{k=1}^d [i_k T,(i_k+1)T]$ and

$$\begin{aligned} \widehat{\mathcal {K}}:= & {} \{{\textbf {i}}=(i_1,...,i_d): 0\leqslant i_k, (i_k-1) T\leqslant \ln (u), k=1,...,d \},\\ \check{\mathcal {K}}:= & {} \{{\textbf {i}}=(i_1,...,i_d): 0\leqslant i_k T\leqslant \ln (u), k=1,...,d \}. \end{aligned}$$

#### Lemma 4.4

There exists a constant $C\in (0,\infty )$ such that for sufficiently large *u*, for all ${\textbf {i}},{\textbf {j}}\in \widehat{\mathcal {K}}, {\textbf {i}}\ne {\textbf {j}}$ we have

$$\begin{aligned} \mathbb {P}{\left\{ \max _{t\in \mathcal {R}_{{\textbf {i}}}} X_u(t)>u,\max _{t\in \mathcal {R}_{{\textbf {j}}}} X_u(t)>u\right\} } \leqslant C T^{2d}\exp \left( -\frac{1}{8} \inf _{t\in \mathcal {R}_{{\textbf {i}}}, s\in \mathcal {R}_{{\textbf {j}}}} \sigma _V^2(t-s) \right) \Psi (u). \end{aligned}$$

#### Proof of Theorem 3.2

The proof consists of two steps, where we find an asymptotic upper and lower bound for the ratio

$$\begin{aligned} \frac{\mathbb {P}{\left\{ \int _{[0,\ln (u)]^d } \mathbbm {I}(X_u(t)>u) dt>x \right\} }}{(\ln (u))^d\Psi (u)}, \end{aligned}$$

as $u\rightarrow \infty$. We note that by Lemma 4.3 the limit, as $u\rightarrow \infty$, of the above fraction is positive and finite.

*Asymptotic upper bound.* If $T>0$, then for sufficiently large *u*

4.10 $$\begin{aligned}{} & {} {\mathbb {P}{\left\{ \int _{[0,\ln (u)]^d } \mathbbm {I}(X_u(t)>u) dt>x \right\} }}\nonumber \\{} & {} \leqslant \mathbb {P}{\left\{ \sum _{{\textbf {i}}\in \widehat{\mathcal {K}}} \int _{\mathcal {R}_{{\textbf {i}}} } \mathbbm {I}(X_u(t)>u) dt>x \right\} }\nonumber \\{} & {} \leqslant \mathbb {P}{\left\{ \exists _{{\textbf {i}}\in \widehat{\mathcal {K}}} \int _{\mathcal {R}_{{\textbf {i}}} } \mathbbm {I}(X_u(t)>u) dt>x \right\} } + \mathbb {P}{\left\{ \exists _{{\textbf {i}},{\textbf {j}}\in \widehat{\mathcal {K}},{\textbf {i}}\ne {\textbf {j}} } \max _{t\in \mathcal {R}_{{\textbf {i}}} } X_u(t)>u, \max _{t\in \mathcal {R}_{{\textbf {j}}} } X_u(t)>u \right\} }\nonumber \\{} & {} \leqslant \sum _{{\textbf {i}}\in \widehat{\mathcal {K}}} \mathbb {P}{\left\{ \int _{\mathcal {R}_{{\textbf {i}}} } \mathbbm {I}(X_u(t)>u) dt>x \right\} } + \mathbb {P}{\left\{ \exists _{{\textbf {i}},{\textbf {j}}\in \widehat{\mathcal {K}},{\textbf {i}}\ne {\textbf {j}} } \max _{t\in \mathcal {R}_{{\textbf {i}}} } X_u(t)>u, \max _{t\in \mathcal {R}_{{\textbf {j}}} } X_u(t)>u \right\} }\nonumber \\{} & {} \leqslant \left\lceil \frac{(\ln (u))^d}{T^d}\right\rceil \mathbb {P}{\left\{ \int _{[0,T]^d } \mathbbm {I}(X_u(t)>u) dt>x \right\} }\nonumber \\ {}{} & {} \quad +\, \mathbb {P}{\left\{ \exists _{{\textbf {i}},{\textbf {j}}\in \widehat{\mathcal {K}},{\textbf {i}}\ne {\textbf {j}} } \max _{t\in \mathcal {R}_{{\textbf {i}}} } X_u(t)>u, \max _{t\in \mathcal {R}_{{\textbf {j}}} } X_u(t)>u \right\} }, \end{aligned}$$

where $\lceil \cdot \rceil$ is the ceiling function and the last inequality above follows from the stationarity of $X_u$. Using again the stationary of $X_u$, we obtain

4.11 $$\begin{aligned}{} & {} {\mathbb {P}{\left\{ \exists _{{\textbf {i}},{\textbf {j}}\in \widehat{\mathcal {K}},{\textbf {i}}\ne {\textbf {j}} } \max _{t\in \mathcal {R}_{{\textbf {i}}} } X_u(t)>u, \max _{t\in \mathcal {R}_{{\textbf {j}}} } X_u(t)>u \right\} }}\nonumber \\{} & {} \leqslant \sum _{{\textbf {i}},{\textbf {j}}\in \widehat{\mathcal {K}},{\textbf {i}}\ne {\textbf {j}} }\mathbb {P}{\left\{ \max _{t\in \mathcal {R}_{{\textbf {i}}} } X_u(t)>u, \max _{t\in \mathcal {R}_{{\textbf {j}}} } X_u(t)>u \right\} }\end{aligned}$$

4.12 $$\begin{aligned}{} & {} \leqslant \left\lceil \frac{(\ln (u))^d}{T^d}\right\rceil \sum _{{\textbf {k}}\in \widehat{\mathcal {K}},{\textbf {k}}\ne {\textbf {0}} }\mathbb {P}{\left\{ \max _{t\in \mathcal {R}_{{\textbf {0}}} } X_u(t)>u, \max _{t\in \mathcal {R}_{{\textbf {k}}} } X_u(t)>u \right\} }\nonumber \\{} & {} = \left\lceil \frac{(\ln (u))^d}{T^d}\right\rceil \left( \sum _{{\textbf {k}}\in \widehat{\mathcal {K}},{\textbf {k}}\ne {\textbf {0}}, \mathcal {R}_{{\textbf {0}}}\cap \mathcal {R}_{{\textbf {k}}}\ne \emptyset }\mathbb {P}{\left\{ \max _{t\in \mathcal {R}_{{\textbf {0}}} } X_u(t)>u, \max _{t\in \mathcal {R}_{{\textbf {k}}} } X_u(t)>u \right\} }\right. \nonumber \\{} & {} \quad +\left. \sum _{{\textbf {k}}\in \widehat{\mathcal {K}},{\textbf {k}}\ne {\textbf {0}}, \mathcal {R}_{{\textbf {0}}}\cap \mathcal {R}_{{\textbf {k}}}=\emptyset } \mathbb {P}{\left\{ \max _{t\in \mathcal {R}_{{\textbf {0}}} } X_u(t)>u, \max _{t\in \mathcal {R}_{{\textbf {k}}} } X_u(t)>u \right\} } \right) \nonumber \\{} & {} =:\left\lceil \frac{\ln ^d(u)}{T^d}\right\rceil \left( \Sigma _1+\Sigma _2\right) . \end{aligned}$$

Next, by Lemma 4.4, for sufficiently large *T*, *u* and some $\text {Const}_{0}>0$

4.13 $$\begin{aligned} \Sigma _2\leqslant & {} C T^{2d} \sum _{{\textbf {k}}\in \widehat{\mathcal {K}},{\textbf {k}}\ne {\textbf {0}}, \mathcal {R}_{{\textbf {0}}}\cap \mathcal {R}_{{\textbf {k}}}=\emptyset }\exp \left( -\frac{1}{8}\sigma _V^2(T{\textbf {k}}) \right) \Psi (u)\nonumber \\\leqslant & {} C T^{2d} \sum _{{\textbf {k}}>{\textbf {0}} } \exp \left( -\text {Const}_{0} T^{\alpha _\infty }\Vert {\textbf {k}}\Vert ^{\alpha _\infty } \right) \Psi (u)\nonumber \\\leqslant & {} {\text {Const}_{1}} T^{2d} \exp \left( - T^{\alpha _\infty /2} \right) \Psi (u). \end{aligned}$$

The upper bound for $\Sigma _1$ follows by a similar argument as used in the proof of Piterbarg (1996, Lem. 6.3), thus we explain only main steps of the argument. For a while, consider the following probability

$$ \mathbb {P}{\left\{ \max _{t\in \mathcal {R}_{{\textbf {0}}} } X_u(t)>u, \max _{t\in \mathcal {R}_{{(1,0,...,0)}} } X_u(t)>u \right\} }. $$

Then, for each $\varepsilon >0$ and sufficiently large *T*, *u*,

4.14 $$\begin{aligned}{} & {} {\mathbb {P}{\left\{ \max _{t\in \mathcal {R}_{{\textbf {0}}} } X_u(t)>u, \max _{t\in \mathcal {R}_{(1,0,...,0)} } X_u(t)>u \right\} }}\nonumber \\{} & {} \leqslant \mathbb {P}{\left\{ \max _{t\in [0,T^\varepsilon ]\times [0,T]^{d-1}} X_u(t)>u \right\} }\nonumber \\ {}{} & {} \quad +\ \mathbb {P}{\left\{ \max _{t\in [0,T]^{d} } X_u(t)>u, \max _{t\in [T^\varepsilon ,T^\varepsilon +T] \times [0,T]^{d-1} } X_u(t)>u \right\} }\nonumber \\{} & {} \leqslant \text {Const}_{2} T^{d-1+\varepsilon }\Psi (u)+\text {Const}_{3} T^{2d}\exp \left( -T^{\varepsilon /2}\right) , \end{aligned}$$

where the above inequality follows by Lemma 4.4 and

$$\begin{aligned}{} & {} \lim _{u\rightarrow \infty }\frac{\mathbb {P}{\left\{ \max _{t\in [0,T^\varepsilon ]\times [0,T]^{d-1}} X_u(t)>u \right\} }}{\Psi (u)}\nonumber \\ {}{} & {} \leqslant \lceil T \rceil ^{(d-1)(1-\epsilon )}\lim _{u\rightarrow \infty } \frac{\mathbb {P}{\left\{ \max _{t\in [0,T^\varepsilon ]^d} X_u(t)>u \right\} }}{\Psi (u)}\\{} & {} =\lceil T \rceil ^{(d-1)(1-\epsilon )} \mathcal {B}_Z([0,T^\varepsilon ]^d,0)\\{} & {} \leqslant \text {Const}_{4} T^{d-1+\varepsilon }, \end{aligned}$$

which is a consequence of the stationarity of $X_u$ and statement (i) of Lemma 4.3 applied to $x=0$. Again, by the stationarity of $X_u$ we can obtain the bound as in (4.14) uniformly for all the summands in $\Sigma _1$.

Application of the bounds (4.12), (4.13), (4.14) to (4.10) leads to the following upper estimate

4.15 $$\begin{aligned}{} & {} {\limsup _{u\rightarrow \infty }\frac{\mathbb {P}{\left\{ \int _{[0,\ln (u)]^d } \mathbbm {I}(X_u(t)>u) dt>x \right\} }}{\ln ^d(u)\Psi (u)}}\nonumber \\{} & {} \leqslant \frac{\mathcal {B}_Z([0,T]^{d},x)}{T^d}\nonumber \\ {}{} & {} \quad +\ \text {Const}_{4}\frac{1}{T^d}\left( T^{d-1+\varepsilon }+T^{2d} \exp \left( - T^{\alpha _\infty /2} \right) +T^{2d}\exp \left( -T^{\varepsilon /2}\right) \right) , \end{aligned}$$

which is valid for all $\varepsilon >0$ and *T* sufficiently large.

*Asymptotic lower bound.* Taking $T>0$, for sufficiently large *u*

4.16 $$\begin{aligned}{} & {} {\mathbb {P}{\left\{ \int _{[0,\ln (u)]^d } \mathbbm {I}(X_u(t)>u) dt>x \right\} }}\nonumber \\{} & {} \geqslant \mathbb {P}{\left\{ \sum _{{\textbf {i}}\in \check{\mathcal {K}}} \int _{\mathcal {R}_{{\textbf {i}}} } \mathbbm {I}(X_u(t)>u) dt>x \right\} }\nonumber \\{} & {} \geqslant \mathbb {P}{\left\{ \exists _{{\textbf {i}}\in \check{\mathcal {K}}} \int _{\mathcal {R}_{{\textbf {i}}} } \mathbbm {I}(X_u(t)>u) dt>x \right\} }\nonumber \\{} & {} \geqslant \sum _{{\textbf {i}}\in \check{\mathcal {K}}}\mathbb {P}{\left\{ \int _{\mathcal {R}_{{\textbf {i}}} } \mathbbm {I}(X_u(t)>u) dt>x \right\} }\nonumber \\ {}{} & {} \quad -\sum _{{\textbf {i}},{\textbf {j}}\in \check{\mathcal {K}},{\textbf {i}}\ne {\textbf {j}} }\mathbb {P}{\left\{ \max _{t\in \mathcal {R}_{{\textbf {i}}} } X_u(t)>u, \max _{t\in \mathcal {R}_{{\textbf {j}}} } X_u(t)>u \right\} }\end{aligned}$$

4.17 $$\begin{aligned}{} & {} \geqslant \left\lfloor \frac{\ln ^d(u)}{T^d}\right\rfloor \mathbb {P}{\left\{ \int _{[0,T]^d } \mathbbm {I}(X_u(t)>u) dt>x \right\} }\nonumber \\ {}{} & {} \quad -\sum _{{\textbf {i}},{\textbf {j}}\in \check{\mathcal {K}},{\textbf {i}}\ne {\textbf {j}} }\mathbb {P}{\left\{ \max _{t\in \mathcal {R}_{{\textbf {i}}} } X_u(t)>u, \max _{t\in \mathcal {R}_{{\textbf {j}}} } X_u(t)>u \right\} }, \end{aligned}$$

where in (4.16) we used Bonferroni inequality.

Using that $\check{\mathcal {K}}\subset \widehat{\mathcal {K}}$ with the upper bound for

4.18 $$\begin{aligned} \sum _{{\textbf {i}},{\textbf {j}}\in \widehat{\mathcal {K}},{\textbf {i}}\ne {\textbf {j}} }\mathbb {P}{\left\{ \max _{t\in \mathcal {R}_{{\textbf {i}}} } X_u(t)>u, \max _{t\in \mathcal {R}_{{\textbf {j}}} } X_u(t)>u \right\} } \end{aligned}$$

derived in (4.11), we conclude that for each *T* sufficiently large and $\varepsilon >0$,

4.19 $$\begin{aligned}{} & {} {\liminf _{u\rightarrow \infty } \frac{\mathbb {P}{\left\{ \int _{[0,\ln (u)]^d } \mathbbm {I}(X_u(t)>u) dt>x \right\} }}{(\ln (u))^d\Psi (u)}}\nonumber \\{} & {} \geqslant \frac{\mathcal {B}_Z([0,T]^{d},x)}{T^d}\nonumber \\ {}{} & {} \quad - \text {Const}_{4}\frac{1}{T^d}\left( T^{d-1+\varepsilon }+T^{2d} \exp \left( - T^{\alpha _\infty /2} \right) +T^{2d}\exp \left( -T^{\varepsilon /2}\right) \right) . \end{aligned}$$

Thus, by statement (ii) of Lemma 4.3 combined with (4.15) and (4.19), in view of the fact that $\varepsilon$ can take any value in (0, 1), we arrive at

$$\begin{aligned} \lim _{T\rightarrow \infty }\left| \mathcal { B}_{ Z }(x)-\frac{\mathcal { B}_{ Z }([0,T]^d,x)}{T^d}\right| T^\lambda =0 \end{aligned}$$

for all $\lambda \in (0,1)$ establishing the proof. $\square$

#### Proof of Proposition 3.4

The idea of the proof is to analyze the asymptotic upper and lower bound of

$$\begin{aligned} \mathbb {P}{\left\{ \epsilon _\delta (Y)>x\right\} } \end{aligned}$$

as $x\rightarrow \infty$ and then to apply Proposition 2.7. In order to simplify the notation, we consider only the case $\delta =0$. Let $Z(t)=V(t)-\sigma ^2_V(t)/2, t\in \mathbb {R}$ with *V* a centered Gaussian process with stationary increments that satisfies **A1-A2** and $\mathcal {W}$ an independent of *V* exponentially distributed rv with parameter 1.

*Logarithmic upper bound.* Let $A\in (0,1/2)$. We begin with an observation that

4.20 $$\begin{aligned}&{\mathbb {P}{\left\{ \epsilon _\delta (Y)>x\right\} }}\nonumber \\&=\mathbb {P}{\left\{ \int _{\mathbb {R}}\mathbbm {I}\{ \mathcal {W}+ V(t)-\sigma ^2_V(t)/2>0 \} dt>x\right\} }\nonumber \\&\leqslant \mathbb {P}{\left\{ \mathcal {W}\leqslant A\sigma ^2_V(x/2), \int _{\mathbb {R}}\mathbbm {I}\{ A\sigma ^2_V(x/2)+ V(t)-\sigma ^2_V(t)/2>0 \} dt>x\right\} }\nonumber \\&\quad +\mathbb {P}{\left\{ \mathcal {W}>A\sigma ^2_V(x/2)\right\} }\nonumber \\&\leqslant e^{-A\sigma ^2_V(x/2)}+\mathbb {P}{\left\{ \int _{\mathbb {R}}\mathbbm {I}\{ A\sigma ^2_V(x/2)+ V(t)-\sigma ^2_V(t)/2>0 \} dt>x\right\} }\nonumber \\&\leqslant e^{-A\sigma ^2_V(x/2)}+\mathbb {P}{\left\{ \sup _{t\in \left( -\infty ,-x/2]\cup [x/2,\infty \right) } V(t)-\sigma ^2_V(t)/2> -A\sigma ^2_V(x/2)\right\} }\nonumber \\&\leqslant e^{-A\sigma ^2_V(x/2)}+2\mathbb {P}{\left\{ \exists _{t\in \left[ x/2,\infty \right) } V(t)>\left( \frac{1}{2} -A\right) \sigma ^2_V(t)\right\} }\end{aligned}$$

4.21 $$\begin{aligned}&= e^{-A\sigma ^2_V(x/2)}+2\mathbb {P}{\left\{ \exists _{t\in \left[ x/2,\infty \right) } \frac{V(t)}{\sigma _V^2(t)}>\left( \frac{1}{2} -A\right) \right\} }\,, \end{aligned}$$

where in (4.20) we used that $\{V(-t),t\geqslant 0\}{\mathop {=}\limits ^{d}} \{V(t),t\geqslant 0\}$ and the assumption that $\sigma ^2_V$ is increasing. Next, by **A1**, for sufficiently large *x* and $s,t\geqslant x/2$ such that $|t-s|\leqslant 1$

$$\begin{aligned} Cov\left( \frac{V(t)}{\sigma _V(t)},\frac{V(s)}{\sigma _V(s)}\right) \geqslant \exp \left( -|t-s|^{\alpha _0/2}\right) =:Cov\left( Z(t),Z(s)\right) , \end{aligned}$$

where *Z* is some centered stationary Gaussian process. Hence, by Slepian inequality (see, e.g., Corollary 2.4 in Adler (1990))

$$\begin{aligned} \mathbb {P}{\left\{ \exists _{t\in \left[ x/2,\infty \right) } \frac{V(t)}{\sigma ^2_V(t)}>\left( \frac{1}{2} -A\right) \right\} }\leqslant & {} \sum _{k=0}^\infty \mathbb {P}{\left\{ \exists _{t\in [x/2+k,x/2+k+1]} \frac{V(t)}{\sigma ^2_V(t)}> \left( \frac{1}{2} -A\right) \right\} }\\\leqslant & {} \sum _{k=0}^\infty \mathbb {P}{\left\{ \exists _{t\in [0,1]} Z(t)> \left( \frac{1}{2} -A\right) {\sigma _V(x/2+k)}\right\} } \end{aligned}$$

and by Landau-Shepp (see, e.g., Adler (1990, Eq. (2.3))), uniformly with respect to *k*

$$\begin{aligned} \lim _{x\rightarrow \infty }\frac{\ln \left( \mathbb {P}{\left\{ \exists _{t\in [0,1]} Z(t)> \left( \frac{1}{2} -A\right) {\sigma _V(x/2+k)}\right\} }\right) }{\sigma _V^2(x/2+k)}=-\frac{1}{2}\left( \frac{1}{2}-A\right) ^2\,. \end{aligned}$$

The above implies that

$$\begin{aligned} \lim _{x\rightarrow \infty }\frac{\ln \left( \mathbb {P}{\left\{ \exists _{t\in \left[ x/2,\infty \right) } \frac{V(t)}{\sigma ^2_V(t)}>\left( \frac{1}{2} -A\right) \right\} }\right) }{\sigma _V^2(x/2)}\leqslant -\frac{1}{2}\left( \frac{1}{2}-A\right) ^2\ . \end{aligned}$$

Thus, in order to optimize the value of *A* in (4.21) it suffices now to solve

$$\begin{aligned} \left( \frac{1}{2}-A\right) ^2=2A \end{aligned}$$

that leads to (recall that $A<1/2$)

$$\begin{aligned} A=\frac{3-2\sqrt{2}}{2}. \end{aligned}$$

Hence

$$\begin{aligned} \lim _{x\rightarrow \infty }\frac{\ln \left( \mathbb {P}{\left\{ \epsilon _\delta (Y)>x\right\} }\right) }{\sigma _V^2(x/2)}\leqslant - \frac{3-2\sqrt{2}}{2}, \end{aligned}$$

which combined with (2.16) in Proposition 2.7 completes the proof of the logarithmic upper bound.

*Logarithmic lower bound.* Taking $A> 1/2$ we have

$$\begin{aligned} \mathbb {P}{\left\{ \epsilon _\delta (Y)>x\right\} }&= \mathbb {P}{\left\{ \int _{\mathbb {R}}\mathbbm {I}\{ \mathcal {W}+ V(t)-\sigma ^2_V(t)/2>0 \} dt>x\right\} }\\&\geqslant \mathbb {P}{\left\{ \mathcal {W}>A\sigma ^2_V(x/2), \int _{\mathbb {R}}\mathbbm {I}\{ A\sigma ^2_V(x/2)+ V(t)-\sigma ^2_V(t)/2>0 \} dt>x\right\} }\\&\geqslant \mathbb {P}{\left\{ \mathcal {W}>A\sigma ^2_V(x/2)\right\} } \mathbb {P}{\left\{ \inf _{t\in [-x/2, x/2]} V(t)> -(A-1/2)\sigma ^2_V(x/2) \right\} }\\&= e^{-A\sigma ^2_V(x/2)} \left( 1-\mathbb {P}{\left\{ \sup _{t\in [-x/2, x/2]} V(t)> (A-1/2)\sigma ^2_V(x/2) \right\} }\right) . \end{aligned}$$

Using that

4.22 $$\begin{aligned}{} & {} {\mathbb {P}{\left\{ \sup _{t\in [-x/2, x/2]} V(t)> (A-1/2)\sigma ^2_V(x/2) \right\} }}\nonumber \\\leqslant & {} 2\sum _{i \in \{0,...,\lfloor x/2 \rfloor -1\}}\mathbb {P}{\left\{ \sup _{t\in [i,i+1]} V(t)> (A-1/2)\sigma ^2_V(x) \right\} }\end{aligned}$$

4.23 $$\begin{aligned}{} & {} +2\mathbb {P}{\left\{ \sup _{t\in [\lfloor {x/2} \rfloor ,{x/2}]} V(t)> (A-1/2)\sigma ^2_V(x) \right\} } \end{aligned}$$

and the fact that by the stationarity of increments of *V*

$$\begin{aligned} \mathbb {E}{\left\{ \sup _{t\in [i,i+1]} V(t)\right\} }=\mathbb {E}{\left\{ \sup _{t\in [i,i+1]} (V(t)-V(i))+V(i)\right\} }=\mathbb {E}{\left\{ \sup _{t\in [0,1]} V(t)\right\} }=:\mu < \infty \end{aligned}$$

we can apply Borell inequality (e.g., Adler (1990, Thm 2.1)) uniformly for all the summands in (4.23) to get that for sufficiently large *x* (recall that $\sigma ^{2}_V$ is supposed to be increasing)

$$\begin{aligned} \mathbb {P}{\left\{ \sup _{t\in [-x/2, x/2]} V(t)> (A-1/2)\sigma ^2_V(x/2) \right\} }\leqslant & {} 4 (x+1)\exp \left( -\frac{((A-1/2)\sigma ^{2}_V(x/2){-}\mu )^2}{2 \sigma ^2_V(x/2)} \right) \\\leqslant & {} \exp \left( - \frac{(A-1/2)^2\sigma ^{2}_V(x/2)}{4}\right) \rightarrow 0 \end{aligned}$$

as $$x\rightarrow \infty $$.

Hence we arrive at

$$\begin{aligned} \liminf _{x\rightarrow \infty }\frac{\ln (\mathbb {P}{\left\{ \epsilon _\delta (Y)>x\right\} })}{\sigma ^2_V(x/2)}\geqslant -A, \end{aligned}$$

which combined with (2.16) in Proposition 2.7 and the fact that, by the proof of the logarithmic upper bound $\mathbb {E}{\left\{ \epsilon _\delta (Y)\right\} }<\infty$ implies

$$\begin{aligned} \liminf _{x\rightarrow \infty }\frac{\ln (\mathcal { B}_{ Z } (x))}{\sigma ^2_V(x)}\geqslant -2A \end{aligned}$$

for all $A>1/2$. This completes the proof. $\square$

## Data Availability

The corresponding author is available for data obtained in the simulations and used for all the tables and graphs.
